# Black-white differences in chronic stress exposures to predict preterm birth: interpretable, race/ethnicity-specific machine learning model

**DOI:** 10.1186/s12884-024-06613-w

**Published:** 2024-06-22

**Authors:** Sangmi Kim, Patricia A. Brennan, George M. Slavich, Vicki Hertzberg, Ursula Kelly, Anne L. Dunlop

**Affiliations:** 1 Nell Hodgson Woodruff School of Nursing, Emory University, Atlanta, GA USA; 2 Department of Psychology, Emory University, Atlanta, GA USA; 3 Department of Psychiatry and Biobehavioral Sciences, University of California, Los Angeles, CA USA; 4 Atlanta VA Health Care System, Atlanta, GA USA; 5 Department of Gynecology and Obstetrics, School of Medicine, Emory University, Atlanta, GA USA

**Keywords:** Chronic stress, Preterm birth, Racial/ethnic disparity, Machine learning, PRAMS

## Abstract

**Background:**

Differential exposure to chronic stressors by race/ethnicity may help explain Black-White inequalities in rates of preterm birth. However, researchers have not investigated the cumulative, interactive, and population-specific nature of chronic stressor exposures and their possible nonlinear associations with preterm birth. Models capable of computing such high-dimensional associations that could differ by race/ethnicity are needed. We developed machine learning models of chronic stressors to both predict preterm birth more accurately and identify chronic stressors and other risk factors driving preterm birth risk among non-Hispanic Black and non-Hispanic White pregnant women.

**Methods:**

Multivariate Adaptive Regression Splines (MARS) models were developed for preterm birth prediction for non-Hispanic Black, non-Hispanic White, and combined study samples derived from the CDC’s Pregnancy Risk Assessment Monitoring System data (2012–2017). For each sample population, MARS models were trained and tested using 5-fold cross-validation. For each population, the Area Under the ROC Curve (AUC) was used to evaluate model performance, and variable importance for preterm birth prediction was computed.

**Results:**

Among 81,892 non-Hispanic Black and 277,963 non-Hispanic White live births (weighted sample), the best-performing MARS models showed high accuracy (AUC: 0.754–0.765) and similar-or-better performance for race/ethnicity-specific models compared to the combined model. The number of prenatal care visits, premature rupture of membrane, and medical conditions were more important than other variables in predicting preterm birth across the populations. Chronic stressors (e.g., low maternal education and intimate partner violence) and their correlates predicted preterm birth only for non-Hispanic Black women.

**Conclusions:**

Our study findings reinforce that such mid or upstream determinants of health as chronic stressors should be targeted to reduce excess preterm birth risk among non-Hispanic Black women and ultimately narrow the persistent Black-White gap in preterm birth in the U.S.

**Supplementary Information:**

The online version contains supplementary material available at 10.1186/s12884-024-06613-w.

## Background

Preterm birth (< 37 weeks’ gestation) has a range of adverse effects on child health, academic, and social outcomes [1], as well as on parents and families (e.g., psychological distress) [2], and generates high educational and medical costs [3]. In 2021, 384,384 infants were born preterm in the U.S [4]. The country’s preterm birth rate rose 4–10.49% in 2021, the highest level reported since at least 2007 [4]. Importantly, the Black-White inequalities in preterm birth have persisted over the years, such that non-Hispanic Black women (14.75%) are approximately 1.5 times more likely than non-Hispanic White women (9.5%) to experience preterm birth [4]. Nevertheless, the underlying causes of this Black-White difference are not fully understood. Although well-established maternal risk factors explain only about half of the PTB risk [5], growing evidence attributes the remaining unexplained risk to chronic stress [1, 6–10].

Study findings are mixed regarding the effects of chronic stress exposures (i.e., chronic stressors) on preterm birth [11, 12]. One contributor to this inconsistency is limitations in study design and modeling to capture the complexities around women’s chronic stressors, which have been conceptualized variably across studies and out of racial/ethnic context [13]. Evidence shows racial/ethnic variations in three common chronic stressors of childbearing-aged women—namely, financial hardship, perceived isolation, and direct/indirect experience of physical violence (e.g., intimate partner violence [IPV]) [14]. These findings suggest that previous conceptualizations of chronic stressors are likely compromised by assuming universal chronic stress experiences across groups defined by race/ethnicity. Moreover, the existing statistical models that included stress as variables assumed linear and independent associations between stressors and between stressors and outcomes. However, such models are less effective in capturing the dynamics of chronic stressors that are synergistic, accumulate over time, and vary in types and effects by race/ethnicity.

To address these evidence gaps, the present study employed a more flexible and sophisticated method—namely, machine learning—for more accurate computation of chronic stressors and subsequent prediction of preterm birth among non-Hispanic Black and non-Hispanic White women in the U.S. Machine learning gives computers the capability to learn without explicit instructions but based on patterns and inference in data [15]. Machine learning is known for its robustness in handling high-dimensional data with many variables combined in non-linear fashions to predict outcomes or detect new patterns in data [16].

In recent decades, an increasing number of studies have used machine learning to predict preterm birth, in which they employed a wide spectrum of machine learning models, from linear regression to deep neural networks [17], and many different data types, including ultrasound imaging, diagnostic screening, fetal monitoring, and genetics [18]. Most of the prior studies used electronic health records data collected from local hospitals, and their variables in the models encompassed a combination of pregnant women’s health conditions, procedures performed at the hospitals, prescriptions, or tests (e.g., bloodwork and ultrasound) [17, 19, 20]. Only a handful used large population data (e.g., national survey, administrative, or birth and death certificate data) that contained variables rich in socioeconomic, psychological, or behavioral factors beyond biomedical factors and were more representative of the population of pregnant women in the U.S [21–24].

Furthermore, prior studies using machine learning focused more on improving model performance than on understanding the implications of those predictions, making the developed machine learning models opaque, not intuitive, or challenging for users to understand. Systems whose decisions cannot be well-interpreted are less likely to be trusted, particularly in healthcare [25]. Hence, there is a critical need to develop interpretable machine learning models that are trustworthy and high-performing in preterm birth prediction, whose outcomes can be reliably used for early identification and intervention with expecting mothers to prevent preterm birth.

This study aimed to (a) develop machine learning models of chronic stress exposures to predict preterm birth risk among non-Hispanic Black women, non-Hispanic White women, and racial/ethnic groups combined; (b) evaluate the models’ prediction accuracy; and (c) identify and compare important features for preterm birth prediction among the three models. To the best of our knowledge, this study is the first to use interpretable machine learning models to investigate how various chronic stressors—along with sociodemographic, medical, and behavioral factors—predict preterm birth among non-Hispanic Black and non-Hispanic White pregnant women in the U.S. in the context of a national, population-based dataset.

## Methods

### Data source

This secondary data analysis used data from the Pregnancy Risk Assessment Monitoring System (PRAMS) linked with birth certificate data collected by the Centers for Disease Control and Prevention (CDC). This study used Phase 7 (2012–2015) and Phase 8 (2016–2017) data, the latest two Phases, for our study findings to reflect as most recent trends as possible. PRAMS is an ongoing, population-based surveillance project established by the CDC to monitor maternal attitudes and experiences (e.g., perceived racial discrimination, stressful live events [SLEs]) before, during, and shortly after pregnancy (CDC, 2022 [26]). Every month, each state participating in the PRAMS selects a sample of newly delivered mothers from live birth certificates by stratified random sampling without replacement (1,300 to 3,400 women each year) to receive a mailed-out questionnaire. The PRAMS questionnaire consists of two parts: core and standard/state-developed questions. The core questionnaire is asked by all participating states, while the standard/state-developed questionnaire is chosen from a pre-tested list of standard questions developed by the CDC or states on their own. As a result, each state’s PRAMS questionnaire is unique, even though most items are shared across states. Questionnaires are mailed between 2 and 4 months after delivery and followed with a telephone interview for non-responders. The final PRAMS dataset is weighted for sample design, nonresponse, and noncoverage to allow the construction of population estimates representative of all women who gave birth in each state during survey years. The CDC PRAMS working group sets a response rate threshold of 55–70% depending on survey years to minimize nonresponse bias [27].

### Inclusion criteria

The analytic sample consisted of first-time mothers who: (a) were aged younger than 50 years at the time of childbirth; (b) delivered live singleton births without birth defects; and (c) identified themselves as non-Hispanic Black or non-Hispanic White. We limited our sample to first-time mothers due to their higher risk of preterm birth than multiparous women [28]. Only birth mothers (not adoptive mothers) were subject to analysis to link birth mothers’ chronic stress exposures to preterm birth. Although the CDC defined 16–49 years as childbearing age, our sample included mothers aged younger than 16 because the maternal age variable in the data was categorical, not allowing us to limit the sample to those aged 16–49 years. Also, only singleton births without birth defects were included because the causes and consequences of adverse birth outcomes in the case of multiple births and birth defects differ from those of singleton births without birth defects.

Originally, the dataset included 222,290 individuals. We excluded those who had a prior live birth (*n* = 32,630), gave birth to twins+ (*n* = 3,504) or an infant with a birth defect (*n* = 3,196), and were not of non-Hispanic Black or non-Hispanic White race/ethnicity (*n* = 54,975). This sequential elimination process reduced the initial sample size to 127,985. The final sample size was 78,356 after deleting missing data. The highest number of missing data was 127,985 observed in two variables (i.e., bleeding during pregnancy and pregnancy complications) (Table S1). With the survey weight considered, the final sample represented 359,855 women, with 81,892 non-Hispanic Black women and 277,963 non-Hispanic White women.

### Measures

**Fig. 1:**
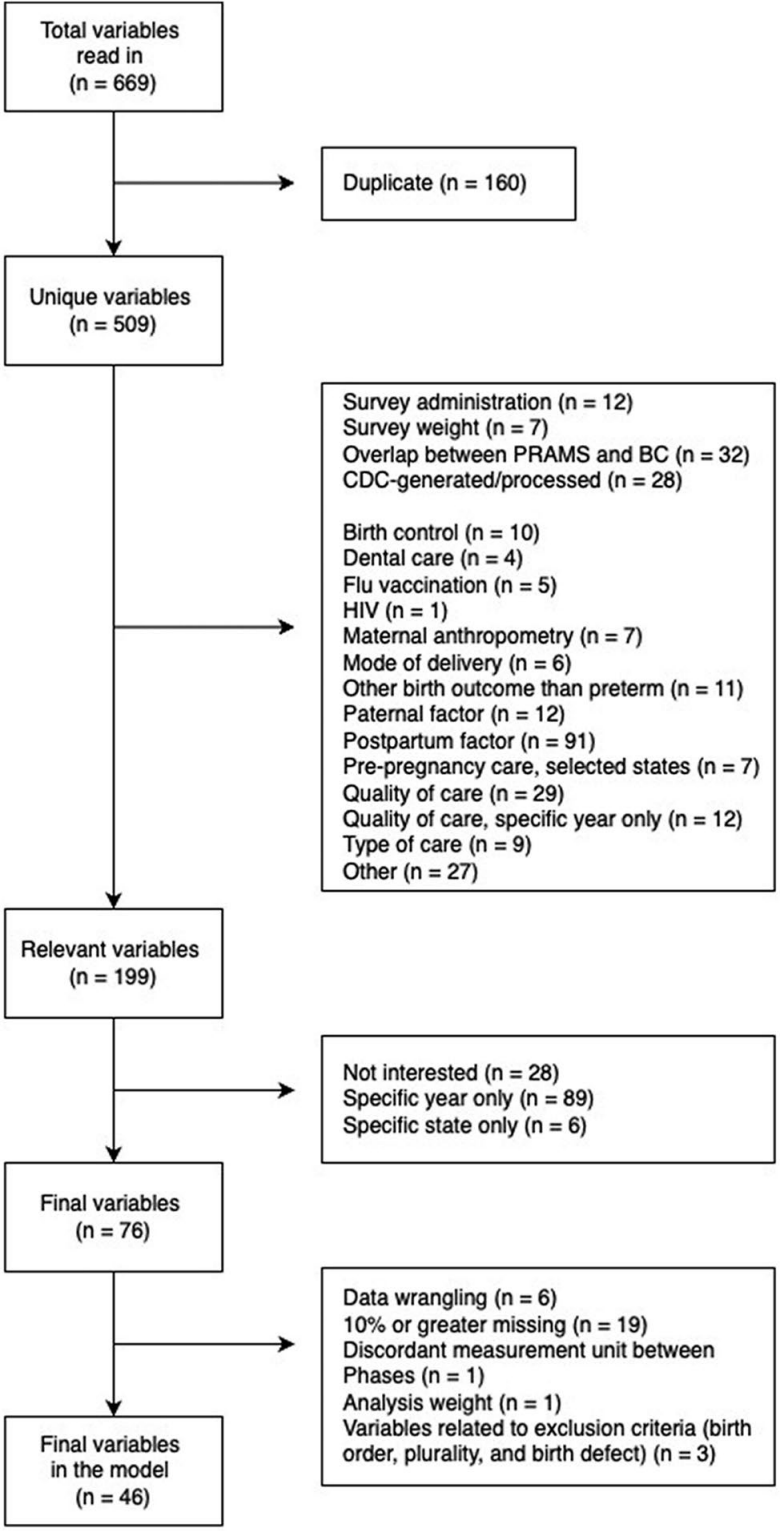
Flow diagram for variable selection

46 out of 669 variables were selected and modeled. Figure 1 is a flow diagram for variable selection. Although the study’s focus was on chronic stressors as predictors for preterm birth, our models included other relevant factors that could mediate or confound the associations between chronic stressors and preterm birth based on the prior literature [7, 11, 29, 30], such as one’s sociodemographic, medical, and behavioral characteristics. Survey years and U.S. states were modeled to factor in potential temporal and spatial variations. However, several variables planned to be analyzed were removed from the analysis due to a substantial amount of missing data (social support, home visitor to help prepare for the new baby, perceived racial discrimination, and perceived neighborhood safety). A comprehensive description of the analyzed variables is provided in Table S2 in the supplemental materials.

#### Preterm birth

The study’s outcome variable was gestational age in completed weeks. This categorical variable ( < = 27, 28–33, 34–36, 37–39, and 40+) was dichotomized into preterm birth (< 37 weeks) and term birth (37 + weeks).

#### Chronic stressors

As external stressors, we analyzed health insurance coverage before and during pregnancy (yes/no), yearly total household income (with 12 levels), maternal educational attainment (0–8, 9–11, 12, 13–15, or 16 + years), receiving WIC (yes/no) as an indication of lower income, physical abuse by a husband/partner before and during pregnancy (yes/no), and 11 items regarding SLEs (yes/no). Some examples of SLEs were separation or divorce, homelessness, arguing with a husband/partner more than usual, and unwanted pregnancy by a husband/partner. As enhancers of stress, we analyzed psychological distress, such as depression before pregnancy (yes/no).

#### Sociodemographic factors

These included maternal race/ethnicity (non-Hispanic Black or non-Hispanic White) and maternal age ( < = 17, 18–19, 20–25, 26–29, 30–34, 35–39, or 40+). For marital status, only two options (married or other) were available in the data.

#### Medical factors

These included the number of pregnancy terminations in the past (as a continuous variable), the presence of pre-pregnancy health conditions (e.g., diabetes mellitus and chronic hypertension), pre-pregnancy body mass index (BMI) (as a continuous variable), gestational diabetes, pregnancy complications (e.g., fever and ruptured membrane), and other medical risk factors. Most of these items were answered yes or no.

#### Behavioral factors

These encompassed multivitamin intake (didn’t take vitamin, 1–3 times/week, 4–6 times/week, or every day/week), pregnancy intention (later, sooner, then, did not want then or any time, or was not sure), the number of prenatal care visits (PNC; <= 8, 9–11, or 12+), initiation of the PNC in the first trimester (yes, no, or no PNC), and the number of cigarettes smoked (before pregnancy, during 1st, 2nd, and 3rd trimester).

### Data analysis

#### Variable selection and handling of missing data

Of the final list of 76 variables associated with chronic stressors and preterm birth, we removed 30 variables during data pre-processing (e.g., merging variables, discarding variables with over 10% missing data [31], and discarding variables neither a predictor nor an outcome) (Fig. 1). However, as an exception, we included variables with a 10-12.2% missing rate to keep the annual household income variable (12.2% missing) in the analysis, as income was an essential indicator of socioeconomic status and a well-known source of chronic stress. By doing so, we automatically included such variables as a cut in work hours or pay of husband/partner/self (11.6% missing) and homeless (11.4% missing). Ultimately, we analyzed 46 variables and 78,356 individuals (359,855 individuals after the application of sampling weight) who met the inclusion criteria and did not have missing data.

#### Descriptive statistics

We investigated the participants’ characteristics and their associations with preterm birth, stratified by race/ethnicity. The characteristics were summarized with frequency, percentage, mean, and standard deviation. Racial/ethnic differences in the characteristics and their associations with preterm birth were examined using Chi-squared tests with Rao & Scott’s second-order correction (for categorical variables) and Wilcoxon rank-sum tests (for continuous variables) for complex survey samples. The statistical significance was set at the alpha level of 5%.

#### Multivariate adaptive regression splines (MARS)

We used a MARS model to predict preterm birth among three groups: non-Hispanic Black women, non-Hispanic White women, and both. MARS is a nonparametric, multivariate regression method that can estimate complex non-linear relations by a series of spline (i.e., piecewise curve) functions of the predictor variables. As a nonparametric approach, MARS does not make any underlying assumptions about the distribution of the predictor variables [32]. MARS considers the relationships between each predictor variable and the outcome variable. For a given predictor variable, MARS partitions across the range of that variable and fits individual linear regression models between partition points. These models are joined at these partition points, also called knots. The process continues through each predictor variable, producing a highly non-linear pattern [33]. Compared to polynomial regression, MARS is more robust at fitting non-linear curves to detect subgroup differences in risk-disease relationships [34]. Importantly, MARS can estimate the relative feature importance via the generalized cross-validation (GCV) [35].

#### Model training and testing

The ratio of training and test set was 70/30. Given the unbalanced data, we partitioned the data in a way that each of the training and test sets contained the same preterm: term birth ratio. We built three models: (a) a baseline model without interactions between the features; (b) a second-degree interaction model; and (c) a third-degree interaction model. We implemented 5-fold cross-validation to select the model with the smallest residual, which was evaluated on the test set later. Although MARS has two tuning parameters—the degree of interactions and the number of retained terms—to minimize prediction error, we needed to tune only the degree of interactions since the cross-validation decided the optimal number of terms for the models. We limited our degree of interactions to three so as not to create an unnecessarily complicated model for the given data and prevent overfitting.

We also analyzed both original and weighted data to develop machine learning models for comparison. The original data were the ones collected by the CDC in a way that mothers of low-birth-weight infants, those living in high-risk geographic areas, and racial/ethnic minority groups were oversampled [27]. The weighted data were the ones that the sampling weight calculated and assigned by the CDC was applied to represent the population of pregnant women who birthed in certain states and survey years. However, how to include sampling weight in machine models is not clearly documented, and developing weighted machine learning models requires extensive computing resources [36]. Therefore, we approximated weighting machine learning models by replicating each observation by the highest integer number of the assigned sampling weight (i.e., converting 5.6 into 5) and training and testing machine learning models on those replicated data. The mean of the sampling weight was 50.94 (range: 1.00–1131.58).

Finally, we calibrated our best-performing model for each population. Specifically, we employed logistic, isotonic, and beta calibration methods for comparison, chose a method generating the best result, and tested the calibrated model on the test set for each population.

#### Model evaluation matrix

We evaluated the performance of each model via the Area Under the Receiver Operating Characteristic (ROC) Curve (AUC). AUC represents the trade-off between the true-positive and the false-positive rates. In ROC analysis, a diagonal identity line starting at zero indicates that output is a random guess, whereas an ideal classifier with a high true-positive rate (sensitivity) and a low false-positive rate (1-specificity) will curve positively and strongly toward the upper left quadrant of the plot.

#### Interpretability

We first created a white-box model, like MARS, and simultaneously interpreted already trained MARS models post hoc [25]. As mentioned earlier, MARS can compute the feature importance via the GCV. For the post hoc analysis, we analyzed our models by assessing the feature importance and feature effect (via partial dependence plot [37] and individual conditional expectation curve [38]). All data analysis was conducted using R version 4.0.2 (2020-06-22).

## Results

### Subject characteristics and associations with preterm birth

Table 1 illustrates maternal characteristics among the *weighted* sample populations. 7.1% of the women experienced preterm birth across racial/ethnic groups, with non-Hispanic Black women being 1.72 times more likely to experience preterm birth than non-Hispanic White women (11% vs. 6.4%). For the original (*unweighted*) sample populations that oversampled high-risk women, 16% of the women experienced preterm birth overall, with non-Hispanic Black women being 1.13 times more likely to experience preterm birth than non-Hispanic White women (17% vs. 15%) (Table S3).

**Table 1:** Sample characteristics by maternal race/ethnicity among weighted sample populations

	Overall N = 51023661^1^	N-H Black N = 764824^1^	N-H White N = 4337542^1^	*p-*value^2^
**Maternal Age**				< 0.001
<= 17	33369 (0.7%)	10346 (1.4%)	23023 (0.5%)	
18–19	148371 (2.9%)	33159 (4.3%)	115212 (2.7%)	
20–24	939993 (18%)	203869 (27%)	736124 (17%)	
25–29	1580646 (31%)	230436 (30%)	1350210 (31%)	
30–34	1569070 (31%)	176838 (23%)	1392233 (32%)	
35–39	696675 (14%)	89073 (12%)	607602 (14%)	
40+	134243 (2.6%)	21104 (2.8%)	113138 (2.6%)	
**Marital Status**				< 0.001
Married	3414596 (67%)	236050 (31%)	3178547 (73%)	
Not Married	1687770 (33%)	528774 (69%)	1158995 (27%)	
**Health Insurance During Pregnancy**				< 0.001
Insured	4534541 (89%)	653614 (85%)	3880928 (89%)	
Uninsured	567825 (11%)	111211 (15%)	456614 (11%)	
**Total Annual Income** ^**3**^				< 0.001
$0 to $15000 (Lv 01)	847105 (17%)	292570 (38%)	554535 (13%)	
$15001 to $19000 (Lv 02)	329022 (6.4%)	86472 (11%)	242549 (5.6%)	
$19001 to $22000 (Lv 03)	237548 (4.7%)	59926 (7.8%)	177621 (4.1%)	
$22001 to $26000 (Lv 04)	208570 (4.1%)	45303 (5.9%)	163267 (3.8%)	
$26001 to $29000 (Lv 05)	184101 (3.6%)	37864 (5.0%)	146237 (3.4%)	
$29001 to $37000 (Lv 06)	314157 (6.2%)	52443 (6.9%)	261714 (6.0%)	
$37001 to $44000 (Lv 07)	273412 (5.4%)	34875 (4.6%)	238536 (5.5%)	
$44001 to $52000 (Lv 08)	289191 (5.7%)	30036 (3.9%)	259155 (6.0%)	
$52001 to $56000 (Lv 09)	165380 (3.2%)	14779 (1.9%)	150601 (3.5%)	
$56001 to $67000 (Lv 10)	313193 (6.1%)	24983 (3.3%)	288210 (6.6%)	
$67001 to $79000 (Lv 11)	334305 (6.6%)	20758 (2.7%)	313547 (7.2%)	
$79001 or more (Lv 12)	1606384 (31%)	64813 (8.5%)	1541571 (36%)	
**No. of Household Members**	2.93 (1.31)	2.82 (1.50)	2.95 (1.27)	< 0.001
**Maternal Education (Years)**				< 0.001
00–08	40884 (0.8%)	6384 (0.8%)	34500 (0.8%)	
09–11	294613 (5.8%)	82448 (11%)	212166 (4.9%)	
12	1053795 (21%)	234064 (31%)	819731 (19%)	
13–15	1572153 (31%)	292205 (38%)	1279948 (30%)	
16+	2140921 (42%)	149724 (20%)	1991198 (46%)	
**Receive WIC During Pregnancy**	1615,470 (32%)	472636 (62%)	1142834 (26%)	< 0.001
**Physical Abuse Before Pregnancy**	107874 (2.1%)	29217 (3.8%)	78656 (1.8%)	< 0.001
**Physical Abuse During Pregnancy**	89971 (1.8%)	26099 (3.4%)	63872 (1.5%)	< 0.001
**Divorce**	295107 (5.8%)	86415 (11%)	208692 (4.8%)	< 0.001
**Homeless**	105203 (2.1%)	38604 (5.0%)	66598 (1.5%)	< 0.001
**Job Loss of Husband/Partner**	516777 (10%)	106897 (14%)	409880 (9.4%)	< 0.001
**Job Loss of Self**	444254 (8.7%)	140125 (18%)	304129 (7.0%)	< 0.001
**Cut in Work Hours or Pay of Husband/Partner/Self**	847054 (17%)	158760 (21%)	688295 (16%)	< 0.001
**Argument More Than Usual**	1035870 (20%)	255504 (33%)	780367 (18%)	< 0.001
**Unwanted Pregnancy by Husband/Partner**	322644 (6.3%)	89109 (12%)	233535 (5.4%)	< 0.001
**Problem Paying Bill**	911910 (18%)	199053 (26%)	712857 (16%)	< 0.001
**Imprisonment of Husband/Partner/Self**	164429 (3.2%)	49153 (6.4%)	115276 (2.7%)	< 0.001
**Problem with Drinking/Drugs of People Close to Me**	611947 (12%)	81,200 (11%)	530,746 (12%)	< 0.001
**Death of People Close to Me**	906658 (18%)	171263 (22%)	735395 (17%)	< 0.001
**Depression Before Pregnancy**	608339 (12%)	70390 (9.2%)	537950 (12%)	< 0.001
**Termination of Pregnancy**	0.46 (0.91)	0.64 (1.11)	0.43 (0.87)	< 0.001
**Diabetes Before Pregnancy**	121338 (2.4%)	22344 (2.9%)	98994 (2.3%)	< 0.001
**Hypertension Before Pregnancy**	238796 (4.7%)	65246 (8.5%)	173550 (4.0%)	< 0.001
**BMI Before Pregnancy**	26.44 (6.59)	28.43 (7.39)	26.10 (6.38)	< 0.001
**Gestational Diabetes**	394879 (7.7%)	71460 (9.3%)	323419 (7.5%)	< 0.001
**Fever During Pregnancy**	70550 (1.4%)	12976 (1.7%)	57574 (1.3%)	0.018
**Medical Risks**				< 0.001
No Risks	4211132 (83%)	597430 (78%)	3613702 (83%)	
Risks	891235 (17%)	167394 (22%)	723840 (17%)	
**Premature Rupture of Membrane**	215658 (4.2%)	35874 (4.7%)	179784 (4.1%)	0.036
**Intake of Multivitamin (Times/Week)**				< 0.001
0	2466095 (48%)	501236 (66%)	1964858 (45%)	
1–3	384794 (7.5%)	66613 (8.7%)	318181 (7.3%)	
4–6	370484 (7.3%)	33950 (4.4%)	336535 (7.8%)	
7	1880993 (37%)	163025 (21%)	1717968 (40%)	
**Pregnancy Intention**				< 0.001
Later	1030697 (20%)	231022 (30%)	799676 (18%)	
Not Sure	716908 (14%)	162222 (21%)	554686 (13%)	
Not Want	299603 (5.9%)	90558 (12%)	209045 (4.8%)	
Sooner	762903 (15%)	66234 (8.7%)	696669 (16%)	
Then	2292255 (45%)	214788 (28%)	2077467 (48%)	
**Start of PNC in 1st Trimester**				< 0.001
No	541979 (11%)	144229 (19%)	397749 (9.2%)	
No PNC	25639 (0.5%)	6802 (0.9%)	18837 (0.4%)	
Yes	4534749 (89%)	613793 (80%)	3920956 (90%)	
**No. of PNC Visits**				< 0.001
<= 08	765111 (15%)	190340 (25%)	574771 (13%)	
09–11	1569790 (31%)	234170 (31%)	1335620 (31%)	
12+	2767465 (54%)	340315 (44%)	2427151 (56%)	
**No. Cigarettes Before Pregnancy**	1.77 (5.85)	0.97 (4.46)	1.91 (6.05)	< 0.001
**No. Cigarettes in 1st Trimester**	1.04 (4.13)	0.56 (2.85)	1.13 (4.31)	< 0.001
**No. Cigarettes in 2nd Trimester**	0.77 (3.39)	0.39 (2.21)	0.84 (3.55)	< 0.001
**No. Cigarettes in 3rd Trimester**	0.68 (3.16)	0.33 (2.00)	0.75 (3.32)	< 0.001
**Gestational Age**				< 0.001
<= 27	20348 (0.4%)	8431 (1.1%)	11917 (0.3%)	
28–33	67803 (1.3%)	19264 (2.5%)	48540 (1.1%)	
34–36	271703 (5.3%)	54197 (7.1%)	217507 (5.0%)	
37+	4742511 (93%)	682932 (89%)	4059579 (94%)	
**Preterm Birth**	359855 (7.1%)	81892 (11%)	277963 (6.4%)	< 0.001

*Note.* PNC = prenatal care, WIC = Special Supplemental Nutrition Program for Women, Infants, and Children

^1^ *n* (%) for categorical variables and mean (*SD*) for continuous variables

^2^ Chi-squared tests with Rao & Scott’s second-order correction (for categorical variables) and Wilcoxon rank-sum tests (for continuous variables) were conducted for complex survey samples

^3^ The total incomes shown in the table indicate values only from the Phase 7 data. The Phase 8 data have different values (slightly higher than those from Phase 7) in each category after taking the inflation into account. However, both Phases have 12 income categories, which were entered into the models as income tiers. The adjusted amount of income under each category from Phase 8 can be found in Table S2

Non-Hispanic Black women were inclined to give birth younger and not in a marital relationship. Relative to non-Hispanic White women, non-Hispanic Black women had worse socioeconomic (e.g., lower income and education), psychological (e.g., more exposure to physical abuse by the partner and SLEs), medical (e.g., higher rates of diabetes and hypertension before pregnancy), and behavioral risk profiles (e.g., unintended pregnancy and fewer PNC visits), with three exceptions: non-Hispanic White women were more likely to have people close to them with drinking/drug problems, to experience depression before pregnancy, and to smoke before and during pregnancy. Similar patterns were observed in the weighted (*replicated*) data (Table S4).

Table 2 presents the *weighted* preterm birth rates by maternal characteristics. Given the same distribution characteristics, non-Hispanic Black women were generally more likely to experience preterm birth than their non-Hispanic White counterparts. We observed the state-level variations in preterm birth rate within and between the racial/ethnic groups (data not shown). We found a maternal age trajectory of preterm birth rate distinct to each racial/ethnic group, in which non-Hispanic Black women showed a maternal age-related increase in preterm birth rate (known as weathering), whereas non-Hispanic White women showed a typical U-shaped pattern with a higher preterm birth rate on the extremes of maternal age with a nadir in 30–34 years of age.

**Table 2:** Number and rates of preterm birth by maternal characteristics among weighted sample populations

	Overall *N* = 359,855^1,2^	N-H Black *N* = 81,892^1,2^	N-H White *N* = 277,963^1,2^
**Maternal Age**			
<= 17	2920 (8.8%)	732 (7.1%)	2188 (9.5%)
18–19	13,711 (9.2%)	3747 (11%)	9964 (8.6%)
20–24	66,105 (7.0%)	20,150 (9.9%)	45,955 (6.2%)
25–29	107,660 (6.8%)	23,362 (10%)	84,298 (6.2%)
30–34	105,142 (6.7%)	19,931 (11%)	85,211 (6.1%)
35–39	51,019 (7.3%)	11,013 (12%)	40,006 (6.6%)
40+	13,298 (9.9%)	2956 (14%)	10,342 (9.1%)
**Marital Status** ^**3**^			
Married	211,632 (6.2%)	23,645 (10%)	187,987 (5.9%)
Not Married	148,223 (8.8%)	58,247 (11%)	89,976 (7.8%)
**Health Insurance Before Pregnancy** ^**3**^			
Insured	310,991 (6.9%)	69,558 (11%)	241,433 (6.2%)
Uninsured	48,864 (8.6%)	12,334 (11%)	36,530 (8.0%)
**Total Annual Income** ^**3**,4^			
$0 to $15,000 (Lv 01)	80,681 (9.5%)	34,067 (12%)	46,614 (8.4%)
$15,001 to $19,000 (Lv 02)	27,567 (8.4%)	9577 (11%)	17,989 (7.4%)
$19,001 to $22,000 (Lv 03)	19,366 (8.2%)	5421 (9.0%)	13,945 (7.9%)
$22,001 to $26,000 (Lv 04)	16,451 (7.9%)	4357 (9.6%)	12,094 (7.4%)
$26,001 to $29,000 (Lv 05)	12,848 (7.0%)	3972 (10%)	8876 (6.1%)
$29,001 to $37,000 (Lv 06)	23,623 (7.5%)	5855 (11%)	17,768 (6.8%)
$37,001 to $44,000 (Lv 07)	18,971 (6.9%)	2805 (8.0%)	16,166 (6.8%)
$44,001 to $52,000 (Lv 08)	18,485 (6.4%)	3163 (11%)	15,322 (5.9%)
$52,001 to $56,000 (Lv 09)	10,023 (6.1%)	1645 (11%)	8379 (5.6%)
$56,001 to $67,000 (Lv 10)	20,750 (6.6%)	2636 (11%)	18,114 (6.3%)
$67,001 to $79,000 (Lv 11)	18,518 (5.5%)	2675 (13%)	15,843 (5.1%)
$79,001 or more (Lv 12)	92,572 (5.8%)	5718 (8.8%)	86,853 (5.6%)
**No. of Household Members** ^**3**^	2.90 (1.39)	2.87 (1.68)	2.90 (1.29)
**Maternal Education (Years)**			
00–08	3177 (7.8%)	718 (11%)	2459 (7.1%)
09–11	29,344 (10.0%)	10,410 (13%)	18,934 (8.9%)
12	89,849 (8.5%)	26,479 (11%)	63,370 (7.7%)
13–15	115,214 (7.3%)	31,186 (11%)	84,028 (6.6%)
16+	122,271 (5.7%)	13,099 (8.7%)	109,172 (5.5%)
**Receive WIC During Pregnancy** ^**3**^	134,304 (8.3%)	49,328 (10%)	84,976 (7.4%)
**Physical Abuse Before Pregnancy** ^**3**^	10,551 (9.8%)	3060 (10%)	7491 (9.5%)
**Physical Abuse During Pregnancy** ^**3**^	8496 (9.4%)	3058 (12%)	5439 (8.5%)
**Divorce** ^**3**^	30,200 (10%)	10,438 (12%)	19,762 (9.5%)
**Homeless** ^**3**^	10,396 (9.9%)	3677 (9.5%)	6719 (10%)
**Job Loss of Husband/Partner** ^**3**^	41,162 (8.0%)	11,280 (11%)	29,882 (7.3%)
**Job Loss of Self** ^**3**^	43,115 (9.7%)	16,674 (12%)	26,442 (8.7%)
**Cut in Work Hours or Pay of Husband/Partner/Self** ^**3**^	67,797 (8.0%)	17,404 (11%)	50,393 (7.3%)
**Argument More Than Usual** ^**3**^	81,590 (7.9%)	26,285 (10%)	55,304 (7.1%)
**Unwanted Pregnancy by Husband/Partner** ^**3**,5^	25,334 (7.9%)	10,106 (11%)	15,228 (6.5%)
**Problem Paying Bill** ^**3**^	75,347 (8.3%)	23,043 (12%)	52,305 (7.3%)
**Imprisonment of Husband/Partner/Self** ^**3**^	17,354 (11%)	5101 (10%)	12,253 (11%)
**Problem with Drinking/Drugs of People Close to Me** ^**3**^	48,608 (7.9%)	9224 (11%)	39,385 (7.4%)
**Death of People Close to Me** ^**3**^	73,195 (8.1%)	19,520 (11%)	53,675 (7.3%)
**Depression Before Pregnancy**	55,751 (9.2%)	9589 (14%)	46,162 (8.6%)
**Termination of Pregnancy**	0.57 (1.06)	0.79 (1.35)	0.50 (0.95)
**Diabetes Before Pregnancy**	16,412 (14%)	3942 (18%)	12,471 (13%)
**Hypertension Before Pregnancy**	35,143 (15%)	12,846 (20%)	22,297 (13%)
**BMI Before Pregnancy**	27.50 (7.40)	29.05 (8.00)	27.05 (7.16)
**Gestational Diabetes** ^**3**^	40,229 (10%)	8265 (12%)	31,964 (9.9%)
**Fever During Pregnancy** ^5^	5879 (8.3%)	1919 (15%)	3960 (6.9%)
**Medical Risk Factors**			
No Risks	228,094 (5.4%)	49,195 (8.2%)	178,899 (5.0%)
Risks	131,761 (15%)	32,697 (20%)	99,064 (14%)
**Premature Rupture of Membrane**	60,085 (28%)	14,768 (41%)	45,317 (25%)
**Intake of Multivitamin (Times/Week)** ^**3**^			
0	184,289 (7.5%)	53,586 (11%)	130,703 (6.7%)
1–3	24,898 (6.5%)	6886 (10%)	18,012 (5.7%)
4–6	20,324 (5.5%)	2730 (8.0%)	17,594 (5.2%)
7	130,344 (6.9%)	18,690 (11%)	111,654 (6.5%)
**Pregnancy Intention** ^**3**^			
Later	76,311 (7.4%)	23,450 (10%)	52,861 (6.6%)
Not Sure	58,362 (8.1%)	17,743 (11%)	40,619 (7.3%)
Not Want	26,408 (8.8%)	10,432 (12%)	15,976 (7.6%)
Sooner	55,305 (7.2%)	8102 (12%)	47,203 (6.8%)
Then	143,469 (6.3%)	22,165 (10%)	121,304 (5.8%)
**Start of PNC in 1st Trimester**			
No	43,452 (8.0%)	16,669 (12%)	26,783 (6.7%)
No PNC	4567 (18%)	1813 (27%)	2754 (15%)
Yes	311,835 (6.9%)	63,409 (10%)	248,426 (6.3%)
**No. of PNC Visits**			
<= 08	142,584 (19%)	39,917 (21%)	102,667 (18%)
09–11	114,511 (7.3%)	19,640 (8.4%)	94,872 (7.1%)
12+	102,760 (3.7%)	22,335 (6.6%)	80,425 (3.3%)
**No. Cigarettes Before Pregnancy**	2.15 (6.07)	1.36 (4.89)	2.38 (6.36)
**No. Cigarettes in 1st Trimester**	1.38 (4.74)	0.82 (3.30)	1.54 (5.07)
**No. Cigarettes in 2nd Trimester**	1.06 (3.78)	0.60 (2.73)	1.19 (4.03)
**No. Cigarettes in 3rd Trimester**	0.93 (3.51)	0.49 (2.45)	1.05 (3.76)

Note. PNC = prenatal care, WIC = Special Supplemental Nutrition Program for Women, Infants, and Children

^1^*n* (%) for categorical variables and mean (*SD*) for continuous variables

^2^ Chi-squared tests with Rao & Scott’s second-order correction (for categorical variables) and Wilcoxon rank-sum tests (for continuous variables) were conducted for complex survey samples

^3^ There was no significant association between preterm birth and marital status, health insurance before pregnancy, total annual income, number of household members, WIC during pregnancy, physical abuse before and during pregnancy, stressful life events, gestational diabetes, intake of multivitamins, and pregnancy intention among the non-Hispanic Black population

^4^ The total incomes shown in the table indicate values only from the Phase 7 data. The Phase 8 data have different values (slightly higher than those from Phase 7) in each category after taking the inflation into account. However, both Phases have 12 income categories, which were entered into the models as income tiers. The adjusted amount of income under each category from Phase 8 can be found in Table S2

^5^ There was no significant association between preterm birth and unwanted pregnancy by husband/partner and fever during pregnancy among the pooled and non-Hispanic White populations

Preterm birth was significantly associated with all risk factors except for unwanted pregnancy by husband/partner and fever during pregnancy for non-Hispanic White women. On the other hand, a smaller set of risk factors—shorter duration of maternal education (i.e., < 16 + years), adverse health outcomes before pregnancy (i.e., depression, diabetes, and hypertension), fever during pregnancy, medical risk factors, premature rupture of membrane (PROM), absent or delayed PNC, and fewer numbers of PNC—increased preterm birth risk for non-Hispanic Black women.

For continuous variables, non-Hispanic Black women with preterm birth experienced more terminations of pregnancy in the past and had higher pre-pregnancy BMI than their non-Hispanic White counterparts. In contrast, non-Hispanic White women with preterm birth were 1.75–2.14 times more likely than their non-Hispanic Black counterparts to smoke before and during pregnancy. Similar patterns were observed in the original (unweighted) (Table S5) and weighted (replicated) data (Table S6).

### Model performance and calibration

We compared the unweighted and weighted (replicated) models according to the study population (pooled, non-Hispanic Black, and non-Hispanic White), interaction (no interaction, 2-way interaction, and 3-way interaction), and dataset (training and test) (Table 3). Each best-performing model had a different number of terms selected by MARS to produce the smallest model errors.

**Table 3:** Preterm risk prediction accuracy by model characteristic

	Training			Testing
	No Interaction	2-way Interaction	3-way Interaction	
	Unweighted^1^			
Pooled	0.814 (0.809–0.819)	**0.815** **(0.810–0.820)**	0.815 (0.810–0.820)	0.813 (0.805–0.820)
N-H Black	0.816 (0.806–0.820)	**0.820** **(0.810–0.830)**	0.817 (0.807–0.827)	0.799 (0.783–0.815)
N-H White	0.814 (0.809–0.820)	**0.815** **(0.809–0.821)**	0.815 (0.809–0.821)	0.814 (0.805–0.823)
	Weighted^2^			
Pooled	0.755 (0.754–0.756)	0.757 (0.756–0.758)	**0.758** **(0.757–0.759)**	0.757 (0.756–0.759)
N-H Black	0.737 (0.734–0.739)	0.751 (0.748–0.753)	**0.757** **(0.754–0.759)**	0.754 (0.750–0.757)
N-H White	**0.765** **(0.764–0.767)**	0.759 (0.758–0.760)	0.759 (0.758–0.760)	0.765 (0.763–0.766)

Note. Bold indicates the highest prediction accuracy within each of the population-specific models

^1^ Unweighted data denote the original survey data that oversampled women with higher risk

^2^ Weighted data are technically pseudo-weighted, mimicking the inclusion of sampling weight in the models by replicating each observation in the data by the highest integer value of the weight variable assigned to each observation

The weighted (replicated) models differed from the unweighted models in their prediction accuracy and best-performing model. Overall, the accuracy of the weighted (replicated) models was lower than the unweighted models across the different modeling conditions. The weighted (replicated) models performed the best with 3-way interactions among the pooled (AUC = 0.758) and non-Hispanic Black populations (AUC = 0.757) and with no interactions among the non-Hispanic White population (AUC = 0.765). When evaluated on the test set, the accuracy of the three models was maintained. The accuracy of the calibrated models, whether unweighted or weighted, was identical to that of the uncalibrated models in our study (Table S7).

### Feature importance and effect

**Fig. 2:**
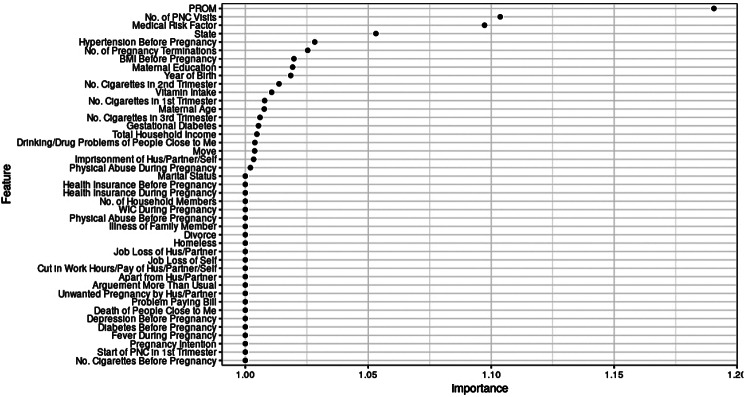
Feature importance in preterm birth risk prediction among N-H black women (weighted/replicated data)

**Fig. 3:**
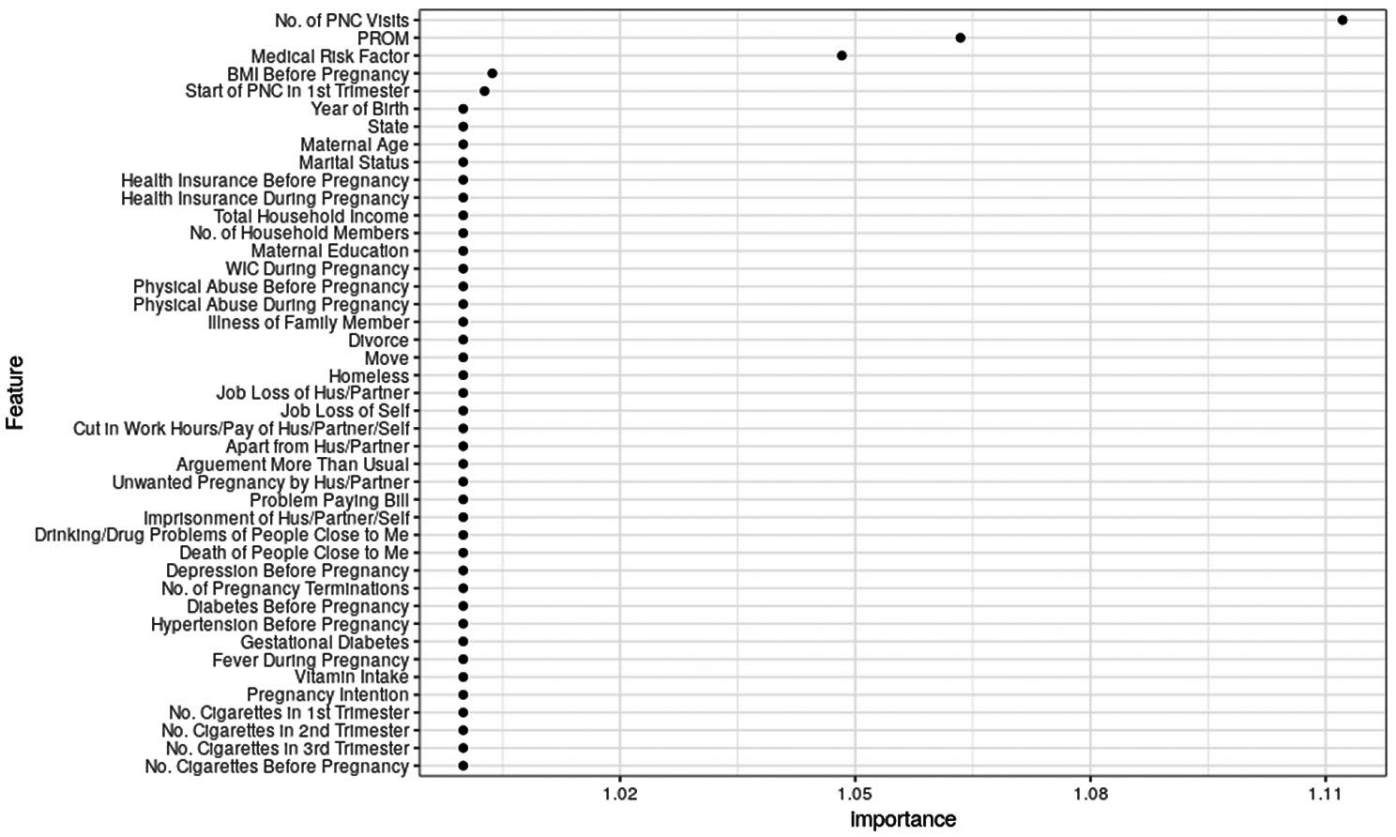
Feature importance in preterm birth risk prediction among N-H white women (weighted/replicated data)

We identified important features in predicting preterm birth risk among non-Hispanic Black and non-Hispanic White women using two different methods (i.e., GCV-based vs. permutation-based) from the weighted (replicated) data for generalizability (Table 4; Fig. 2, and Fig. 3). Important features from the unweighted data can be found in the supplemental materials (Figure S1 and Figure S2). Despite some variations, both methods generally came to the same conclusion. We found important features in common and distinct to each racial/ethnic group.

**Table 4:** Generalized cross-validation-based variable importance of the best-performing models with weighted (replicated)^1^ data

Rank	Pooled	N-H Black	N-H White
1	PROM (Yes)	PROM (Yes)	PROM (Yes)
2	Medical risks (Yes)	Medical risks (Yes)	No. of PNC visits (12+)
3	No. of PNC visits (12+)	No. of PNC visits (12+)	Medical risks (Yes)
4	No. of PNC visits (9–11)	Smoking before pregnancy	No. of PNC visits (9–11)
5	Diabetes before pregnancy	No. of PNC visits (9–11)	Initiation of PNC in the 1st trimester
6	Smoking in the 2nd trimester	Hypertension before pregnancy	BMI before pregnancy
7	MA	BMI before pregnancy	
8	Hypertension before pregnancy	Physical abuse during pregnancy	
9	GA	Smoking in the 2nd trimester	
10		No. of pregnancy termination	
11		Maternal age (30–34 years)	
12		Maternal education (16 + years)	
13		No. of vitamin intake (7 times/week)	
14		Gestational diabetes	
15		Birth year (2017)	
16		GA	
17		Imprisonment of husband/partner/self	
18		Maternal education (12 years)	
19		Move to a new address	
20		CO	
21		Birth year (2013)	
22		Receiving WIC (Yes)	
23		LA	
24		Unwanted pregnancy	
25		Income (level 7)	
26		Smoking in the 3rd trimester	

Note. BMI = Body Mass Index, CO = Colorado, GA = Georgia, LA = Louisiana, MA = Massachusetts, PNC = Prenatal Care, PROM = Premature Rupture of Membrane

^1^ Weighted data are technically pseudo-weighted, mimicking the inclusion of sampling weight in the models by replicating each observation in the data by the highest integer value of the weight variable assigned to each observation

Specifically, the number of PNC visits, PROM, and medical risk factors were the top three important features across the racial/ethnic groups, although their degrees of importance varied according to the method applied and the study population. In addition, non-Hispanic Black women had more important features for preterm birth prediction than non-Hispanic White women (26 vs. 6 important features via GCV). Unlike non-Hispanic White women, the important features of non-Hispanic Black women included a range of chronic stressors, such as physical abuse during pregnancy, maternal education, SLEs (i.e., imprisonment of husband/partner/self, move to a new address), and household income. Moreover, hypertension before pregnancy, states (i.e., GA, CO, and LA), history of pregnancy termination, BMI before pregnancy, multivitamin intake, and smoking in the second and third trimesters during pregnancy were identified as important among non-Hispanic Black women. Most of these factors are known to be associated with chronic stress. On the other hand, the initiation of PNC in the first trimester and BMI before pregnancy were identified as important among non-Hispanic White women.

For the effects of the top three important features, the predicted probability of preterm birth was greater when pregnant women (both racial/ethnic groups) received the lower number of PNC, experienced PROM, and had medical risk factors (Figure S3 and Figure S4).

## Discussion

Although substantial evidence points to robust race/ethnic disparities in preterm birth in the U.S., the drivers of these disparities remain unclear. To address this issue, we built interpretable and race/ethnicity-specific MARS models to predict preterm birth among non-Hispanic Black and non-Hispanic White pregnant women in the U.S. using a large, nationally representative dataset. More specifically, we compared the prediction accuracy between the models with different specifications, as well as with different datasets: original (unweighted) data that oversampled high-risk pregnant women and weighted data more representative of the pregnant women in the U.S. Importantly, we found commonalities and differences in the important features for preterm birth prediction between non-Hispanic Black and non-Hispanic White women. The number of PNC visits, PROM, and medical risk factors were the most important features for both racial/ethnic groups. Only the non-Hispanic Black model identified several chronic stressors and their medical and behavioral correlates as important features for preterm birth prediction, whose findings were masked in the pooled model.

The existing studies have employed a wide spectrum of machine learning models to predict preterm birth, from linear regression to deep learning [20, 39–43]. Despite its strengths that are simple yet sophisticated enough to model the non-linearity and transparent to inform important features for prediction, few studies used MARS models to predict preterm birth among non-Hispanic Black and non-Hispanic White pregnant women.

However, our MARS models performed better than linear models in some prior studies [23, 40], supporting the argument that no simple linear hyperplanes could separate preterm birth from term birth [44]. Moreover, our MARS models’ prediction accuracy was higher than prior studies using different machine learning models and national datasets, including the PRAMS data [21–23].

We observed that approximately 6–7% reduction in AUC from the unweighted to the weighted models on the test set across the study populations. This finding was consistent with that of MacNell and colleagues, who fit gradient boosting models on the National Health and Nutrition Examination Survey data to predict all-cause mortality and reported that the unweighted model performance was inflated compared to the weighted model (F_1_ score: 81.9% vs. 77.4%) [36].

We also found a multitude of important features for preterm birth prediction unique to non-Hispanic Black and non-Hispanic White women. The identified risk factors for preterm birth were evidenced by the existing literature. Both non-Hispanic Black and non-Hispanic White models commonly identified the number of PNC visits [45], PROM [46], and medical conditions [1] as the most robust predictors for preterm birth. Unlike the non-Hispanic White model, however, the non-Hispanic Black model identified an extensive list of predictors that included chronic stressors and their correlates—namely, hypertension before pregnancy [47–49], history of pregnancy termination [50], maternal education [51, 52], maternal BMI [48], multivitamin intake [53], smoking [48, 49, 54], IPV [55, 56], SLEs (i.e., move and imprisonment) [57, 58], gestational diabetes [48, 49], maternal age [11, 49], and household income [59]. In addition, we observed the state-level differences in the predicted preterm birth risk within and across the study populations [60]. The identified important features for non-Hispanic Black women are a reflection of the unjust and racialized social structure in the U.S., such as unequal opportunities and access to individual and neighborhood resources, as well as racial bias in the criminal justice system, which has a trickle-down effect on individual women’s medical conditions, behaviors, and ultimately preterm birth. The country’s extensive current efforts in reducing maternal morbidity and mortality should be directed toward health policies that tackle upstream social determinants of health. In the same vein, healthcare systems should institutionalize policies to address their patients’ social needs to achieve optimal clinical outcomes. Healthcare systems can develop their own programs while there exist multiple resource referral platforms (e.g., findhelp.org) through which healthcare providers can connect their patients to information and referral systems for community resources [61, 62].

Despite some overlaps, however, the current and previous studies showed some variations in the important features for preterm birth prediction. For example, whereas Lee et al. [43] found hypertension, BMI, cervical length, and age, Tran et al. [44] indicated multiple fetuses, cervix incompetence, and prior preterm birth as important features. On the other hand, Gao et al. [20] indicated twin pregnancy, systemic lupus erythematosus, short cervical length, hypertensive disorder, and hydroxychloroquine sulfate. The observed differences can be attributed to the different datasets, models, and analytic populations (e.g., primiparous or multiparous women). Especially, we noticed that the important features in other studies were predominantly represented by biomedical risk factors. In contrast, our study’s important features for non-Hispanic Black women also encompassed various social determinants of health, like chronic stressors, beyond biomedical factors.

The unique sets of important features for preterm birth prediction could be found only because we stratified the data and developed race/ethnicity-specific models. In our data, non-Hispanic White women were disproportionately represented; hence, the non-Hispanic White and the pooled models performed similarly. By stratifying the data according to race/ethnicity, we were able to train our models on the data that had a fair representation of each racial/ethnic group to predict preterm birth with higher accuracy and less bias. Treating race/ethnicity as a marker for differential experiences of and exposure to chronic stress could help overcome limitations of the decontextualized chronic stress models, such as low accuracy or inconclusive association, as the lived experience of each racial/ethnic population is closely linked to their unique cultural, social, regional, and historical contexts, making each population’s experience different from others [63]. Our study findings subscribed to this premise as they showed the predictors of preterm birth that are distinct to each racial/ethnic group and given the same predictor, varying in its magnitude of contribution to preterm birth risk. Moreover, prior studies reported that race/ethnicity-specific machine learning models outperformed race/ethnicity-combined machine learning models [32, 64]. The accuracy of our weighted models mimicked such a pattern, in which the non-Hispanic Black model performed similarly to the pooled model, and the non-Hispanic White model outperformed the pooled model.

All these promising findings of MARS models, or broadly machine learning models, however, should be interpreted and applied with caution since the field, albeit exponentially growing, is still nascent in healthcare. The prediction of preterm birth is bound by the dataset used; hence, the same model can result in different prediction outcomes in different settings with different populations of pregnant women. We should also be vigilant of the potential bias of machine learning models as they can easily overfit data and may not work well in real-world settings, which could harm individuals in the worst-case scenario. Importantly, machine learning can propagate bias in underlying data, producing skewed knowledge and contributing to exacerbated health inequalities. Although MARS is a white box model through which users can learn what factors drove the prediction, many others are black box models, compromising the models’ transparency, interpretability, and trust between developers and users (e.g., patients and healthcare providers).

Further, we should acknowledge numerous challenges to translating machine learning models in research into practice to assist practitioners who serve pregnant women. Examples include the collection of high-quality data, effective data management and data governance strategies, a pipeline for data processing and machine learning with a user-friendly front end, and legal procedures and protection, among others [65]. Therefore, machine learning should be harnessed with balanced views, keeping its promises and perils in mind. We believe that our race/ethnicity-specific, interpretable, and weighted machine learning models using nationally representative data can contribute to the continuation of this important discussion moving forward.

### Limitations

First, our variables in the analysis were limited due to the secondary data. Specifically, we could not predict different subtypes of preterm birth—namely, spontaneous preterm labor, preterm premature rupture of membranes (PPROM), and medically indicated preterm birth since the only variable available was a clinical estimate of gestational age. Consequently, our findings from grouping different subtypes of preterm birth in one may have obscured more nuanced predictors for each subtype of preterm birth, requiring cautious interpretation and application of the study findings. For the same reason, we also included only individual-level factors to predict preterm birth. Although the PRAMS data contained social support, perceived racial discrimination, and perceived neighborhood safety, we excluded them from the analysis due to their high volume of missing data. These variables were collected only by a few states, although they did not always collect them at the same time. If we had modeled these social determinants of health, the current important features and their order to predict preterm birth may have changed. Also, considering that racial discrimination and unsafe neighborhoods are deemed significant risk factors for preterm birth in racial/ethnic minority communities, our non-Hispanic Black model may have underperformed without those variables.

Second, our study inherited limitations of the self-reported measures, such as recall bias, social desirability bias, and response bias stemming from differences in how survey respondents understand/perceive the questions asked.

Third, our weighted models did not directly factor in the survey’s sampling weight for modeling due to the extensive computing power required. To mitigate the problem, we took an alternative approach that replicated each observation by its assigned weight value. It is likely that the prediction outcomes of our pseudo-weighted models deviate from the outcomes of the true weighted models. Nevertheless, the fact that the distribution of maternal characteristics was very similar when directly applying the sampling weight vs. using the replicated data in lieu of the sampling weight alludes to the possibility that the discrepancies in prediction outcomes may not be salient.

Fourth, our input features did not include biological measures, including stress biomarkers. Considering the potential heterogeneity of biological responses to chronic stressors among different racial/ethnic groups of pregnant women, the inclusion of stress biomarkers as input features could improve the prediction accuracy and help us find biomarkers for intervention unique to non-Hispanic Black and non-Hispanic White women. However, our models that predicted preterm birth with demographic, psychosocial, medical, and behavioral factors may be more likely to be used, particularly in under-resourced settings where certain testing, often expensive, and resultant biomarker data are not available.

Lastly, although our models showed high prediction accuracy in general, the accuracy of the preterm birth cases was poor, a finding not uncommon in other studies [66]. One of the main reasons is likely data imbalance, with a far smaller number of preterm birth cases than term birth cases. We did not oversample preterm birth cases nor undersample term birth cases because such data preprocessing could generate artificial data that may have little in common with real observations and infuse bias into the models. Nonetheless, we acknowledge that using the imbalanced data in this study could have undermined the prediction accuracy of preterm birth cases. Future studies will analyze more balanced data using under- and oversampling techniques to investigate variations in prediction accuracy and important features between models with different conditions. In addition to the data imbalance, we suspect a possibility that preterm birth cases did not capture all characteristics of term births or that many preterm birth cases had similar information with term births [20].

## Conclusions

In conclusion, the U.S. continues to experience persistent Black-White inequalities in rates of preterm birth. Although the causes of such inequalities are complex, chronic stress is acknowledged as a highly plausible and potentially major contributor to these inequalities [67]. Therefore, predicting preterm birth and examining the contribution of chronic stress to its prediction are important research directions. Our study further established the role of interpretable, race/ethnicity-specific machine learning models as a useful tool to generate risk prediction systems that could inform key factors behind the preterm prediction unique to non-Hispanic Black and non-Hispanic White pregnant women for targeted prevention and intervention. Although our models did not consider all the risk and protective factors for preterm birth, including biomarkers, we found that multiple chronic stressors and their correlates made a significant and unique contribution to preterm birth prediction among non-Hispanic Black but not non-Hispanic White women, indicating more efforts are called for to tackle the identified chronic stressors (mid or upstream social determinants of health) to alleviate the Black-White inequalities in preterm birth.

Considering that good models can only come from good data, we call for national surveillance systems to collect multi-level social determinants of health beyond the individual level by all U.S. states, as well as outcome variables clinically more meaningful (e.g., subtypes of preterm birth). This can make research findings more applicable or translatable to health policies and practices in the real world to prevent preterm birth among vulnerable women.

Moreover, given the complex and heterogeneous mechanisms underlying preterm birth among different racial/ethnic groups in different geographical and social contexts, the interdisciplinary approach combining data science with traditional epidemiological or qualitative research is critical to shedding light on these mechanisms and tackling inequalities in preterm birth. We find it a promising avenue for future studies to model multi-level determinants of health (from physiological to structural factors) with more powerful machine learning models (e.g., deep learning or hybrid) to predict accurately different subtypes of preterm birth among women with intersecting identities. Especially given that the information on the majority cases (i.e., term birth) tends to drive the model’s overall prediction accuracy, developing machine learning models that can detect preterm birth, separate from term birth, will be an important task.

### Electronic supplementary material

Below is the link to the electronic supplementary material.

Supplementary Material 1

## Data Availability

The data that support the findings of this study are available from the CDC PRAMS but restrictions apply to the availability of these data, which were used upon the CDC PRAMS team’s approval for the current study, and so are not publicly available. Sangmi Kim can be contacted to request the data from this study.

## Abbreviations

AUC: Area Under the Receiver Operating Characteristic Curve
BMI: Body Mass Index
CDC: Centers for Disease Control and Prevention
GCV: Generalized Cross Validation
MARS: Multivariate Adaptive Regression Splines
PNC: Prenatal Care
PRAMS: Pregnancy Risk Assessment Monitoring System
PROM: Premature Rupture of Membrane
ROC: Receiver Operating Characteristics
WIC: Special Supplemental Nutrition Program for Women, Infants, and Children
