# Supplementary material for: Black-white differences in chronic stress exposures to predict preterm birth: interpretable, race/ethnicity-specific machine learning model
Source: BMC Pregnancy Childbirth. 2024 Jun 22;24:438. doi: 10.1186/s12884-024-06613-w (PMC11193905; doi:10.1186/s12884-024-06613-w)

**Table S1**

*Number and Rates of Missing Data for Each Variable (Unweighted Sample)*

| **Variable** | **No. of**  **Missing Data** | **Percentage of Missing Data** |
| --- | --- | --- |
| Bleeding during pregnancy | 127985 | 100.00 |
| Pregnancy complications | 127985 | 100.00 |
| Have someone loaning me money | 113363 | 88.57 |
| Have someone listening to me | 113348 | 88.56 |
| Have someone helping me when I am sick | 113341 | 88.55 |
| Have someone giving me a ride to clinic | 113334 | 88.55 |
| Racial discrimination | 96793 | 75.62 |
| Neighborhood safety | 94889 | 74.14 |
| Acknowledgement of paternity | 76231 | 59.56 |
| Drinking before pregnancy | 70352 | 54.96 |
| Drinking during pregnancy | 70221 | 54.86 |
| Weight change during pregnancy | 51558 | 40.28 |
| Pregnancy history | 48284 | 37.72 |
| Home visitor | 35348 | 27.61 |
| Prescription medicine before pregnancy | 35337 | 27.61 |
| Dieting before pregnancy | 35331 | 27.60 |
| Exercise 3+ days/week before pregnancy | 35317 | 27.59 |
| Saw a dentist for problems | 18418 | 14.39 |
| Needed to see a dentist for problems | 18349 | 14.33 |
| Total annual income | 15600 | 12.18 |
| Cut in work hours or pay of husband/partner/self | 14891 | 11.63 |
| Homeless | 14615 | 11.41 |
| Argument with husband/partner more than usual | 13083 | 10.22 |
| Job loss of husband/partner | 13055 | 10.20 |
| Imprisonment of husband/partner/self | 13018 | 10.17 |
| Unwanted pregnancy by husband/partner | 12994 | 10.15 |
| Apart from husband/partner | 12956 | 10.12 |
| Job loss of self | 12928 | 10.10 |
| Death of people close to me | 12927 | 10.10 |
| Illness of family member | 12924 | 10.09 |
| Problem paying bill | 12902 | 10.08 |
| Problem with drinking/drugs of people close to me | 12849 | 10.03 |
| Move to a new address | 12848 | 10.03 |
| Divorce | 12800 | 10.00 |
| Number of household members | 5266 | 4.11 |
| No. cigarettes smoked before pregnancy | 5048 | 3.94 |
| No. cigarettes smoked in the 1st trimester | 5043 | 3.94 |
| No. cigarettes smoked in the 2nd trimester | 5043 | 3.94 |
| No. cigarettes smoked in the 3^rd^ trimester | 5036 | 3.93 |
| Premature rupture of membrane | 4848 | 3.78 |
| Number of prenatal care visits | 4588 | 3.58 |
| Maternal BMI before pregnancy | 3275 | 2.55 |
| Start of prenatal care in the 1^st^ trimester | 2914 | 2.27 |
| Receive WIC during pregnancy | 2180 | 1.70 |
| Pregnancy intention | 2141 | 1.67 |
| Health insurance during pregnancy | 2004 | 1.56 |
| Physical abuse by a partner/husband during pregnancy | 1876 | 1.46 |
| Physical abuse by a partner/husband before pregnancy | 1853 | 1.44 |
| Gestational diabetes | 1795 | 1.40 |
| Diabetes before pregnancy | 1756 | 1.37 |
| Depression before pregnancy | 1601 | 1.25 |
| High blood pressure before pregnancy | 1556 | 1.21 |
| Number of terminations of pregnancy | 1279 | 0.99 |
| Intake of multivitamin | 1014 | 0.79 |
| Medical risk factors | 893 | 0.69 |
| Maternal educational attainment | 662 | 0.51 |
| Gestational week or preterm birth | 218 | 0.17 |
| Health insurance before pregnancy | 146 | 0.11 |
| Marital status | 87 | 0.06 |
| Fever | 81 | 0.06 |
| Maternal age | 2 | 0.001 |

*Note.*

Year of birth, U.S. state, birth order, plurality, birth defect, and maternal race/ethnicity did not have missing data.

**Table S2**

*Analyzed Variables and Codes*

|  | **Variable Name** | **Description** | **Response Option** |
| --- | --- | --- | --- |
| Metadata | YY_DOB^1^ | Year of birth | 2012-2017 |
|  | STATE^1^ | State name | AK, AL, AR, CO, CT, DE, GA, HI, IA, IL, KS, KY, LA, MA, MD, ME, MI, MN, MO, MT, NC, ND, NE, NH, NJ, NM, NY, OH, OK, OR, PA, PR, RI, SD, TN, TX, UT, VA, VT, WA, WI, WV, WY, YC |
| Birth order | B_ORDER^1^ | Birth order | Numeric |
| Plurality | PLURAL^1^ | Plurality | 1 = SINGLE  2 = TWIN  3 = OTH MULT |
| Birth defect | DEFECT^1^ | Was the baby born with a birth defect? | 1 = YES  2 = NO |
| Ethnicity | HISP_BC^1^ | Hispanic? | 1 = YES  2 = NO |
| Race | MAT_RACE^1^ | Maternal race | 1 = OTH ASIAN  2 = WHITE  3 = BLACK  4 = AM INDIAN  5 = CHINESE  6 = JAPANESE  7 = FILIPINO  8 = HAWAIIAN  9 = OTH-NONWHT  10 = AK NATIVE  11 = MIXED RACE |
| Maternal age | MAT_AGE_NAPHSIS^1^ | Maternal age grouped | 1 = ≤ 17  2 = 18-19  3 = 20-24  4 = 25-29  5 = 30-34  6 = 35-39  7 = 40+ |
| Marital status | MARRIED^1^ | Marital status | 1 = YES  2 = NO |
| Health insurance coverage | BG_INS^2^ | During the month before you got pregnant with your new baby, did you have health insurance? | 1 = NO  2 = YES |
|  | PG_INS^2^ | During your most recent pregnancy, did you have health insurance for your prenatal care? | 1 = NO  2 = YES |
| Yearly total household income | INCOME7 (Phase 7) | Total income 12 months before the most recent pregnancy | 1 = $     0 TO $15,000  2 = $15,001 TO $19,000  3 = $19,001 TO $22,000  4 = $22,001 TO $26,000  5 = $26,001 TO $29,000  6 = $29,001 TO $37,000  7 = $37,001 TO $44,000  8 = $44,001 TO $52,000  9 = $52,001 TO $56,000  10 = $56,001 TO $67,000  11 = $67,001 TO $79,000  12 = $79,001 OR MORE |
|  | INCOME8 (Phase 8) | Total income 12 months before the most recent pregnancy | 1 = $     0 TO $16,000  2 = $16,001 TO $20,000  3 = $20,001 TO $24,000  4 = $24,001 TO $28,000  5 = $28,001 TO $32,000  6 = $32,001 TO $40,000  7 = $40,001 TO $48,000  8 = $48,001 TO $57,000  9 = $57,001 TO $60,000  10 = $60,001 TO $73,000  11 = $73,001 TO $85,000  12 = $85,001 OR MORE |
| Number of dependents | INC_NDEP | Dependents (+self) of the reported total income | Numeric |
| Maternal education | MAT_ED^1^ | Maternal educational attainment | 1 = 0-8 years  2 = 9-11 years  3 = 12 years  4 = 13-15 years  5 = ≥ 16 years |
| Special Supplemental Nutrition Program for Women, Infants, and Children (WIC) | MAT_WIC^1^ | Mother get WIC food during pregnancy? | 1 = YES  2 = NO |
| Intimate partner violence | PAB6HUS | Physical abuse by a husband/partner 12 months before the most recent pregnancy | 1 = NO  2 = YES |
|  | PAD6HUS | Physical abuse by a husband/partner 12 months during the most recent pregnancy | 1 = NO  2 = YES |
| Stressful life event during the 12 months before the new baby was born | STRS_FM3 | A close family member was very sick and had to go into the hospital. | 1 = NO  2 = YES |
|  | STRS_DV3 | I got separated or divorced from my husband or partner. | 1 = NO  2 = YES |
|  | STRS_MOV | I moved to a new address. | 1 = NO  2 = YES |
|  | STRSHOME | I was homeless or had to sleep outside, in a car, or in a shelter. | 1 = NO  2 = YES |
|  | STRS_JOB | My husband or partner lost their job. | 1 = NO  2 = YES |
|  | STRS_WRK | I lost my job even though I wanted to go on working. | 1 = NO  2 = YES |
|  | STRS_PAY | My husband, partner, or I had a cut in work hours or pay. | 1 = NO  2 = YES |
|  | STRS_AWY | I was apart from my husband or partner due to military deployment or extended work-related travel. | 1 = NO  2 = YES |
|  | STRS_ARG | I argued with my husband or partner more than usual. | 1 = NO  2 = YES |
|  | STRS_PG | My husband or partner said they didn’t want me to be pregnant. | 1 = NO  2 = YES |
|  | STRS_BIL | I had problems paying the rent, mortgage, or other bills. | 1 = NO  2 = YES |
|  | STRS_JL3 | My husband, partner, or I went to jail. | 1 = NO  2 = YES |
|  | STRS_DRG | Someone very close to me had a problem with drinking or drugs. | 1 = NO  2 = YES |
|  | STRS_DH3 | Someone very close to me died. | 1 = NO  2 = YES |
| Psychological distress | BPG_DEPRS (Phase 7) | Depression before pregnancy | 1 = NO  2 = YES |
|  | BPG_DEPRS8 (Phase 8) | Depression before pregnancy | 1 = NO  2 = YES |
| Preterm birth | GEST_WK_NAPHSIS^1^ | Clinical estimate of gestational age grouped | 1 = ≤ 27  2 = 28-33  3 = 34-36  4 = 37-42  5 = 43+ |
| Reproductive history | OTH_TERM^1^ | History of pregnancy terminations | Numeric  0 = NONE  7 = 7 OR MORE |
| Physical health before and during pregnancy | BPG_DIAB (Phase 7) | Pre-pregnancy diabetes | 1 = NO  2 = YES |
|  | BPG_DIAB8 (Phase 8) | Pre-pregnancy diabetes | 1 = NO  2 = YES |
|  | PG_GDB (Phase 7) | Gestational diabetes - this pregnancy | 1 = NO  2 = YES |
|  | PG_GDB8 (Phase 8) | Gestational diabetes - this pregnancy | 1 = NO  2 = YES |
|  | BPG_HBP (Phase 7) | Pre-pregnancy high blood pressure | 1 = NO  2 = YES |
|  | BPG_HBP8 (Phase 8) | Pre-pregnancy high blood pressure | 1 = NO  2 = YES |
| Body mass index | MOM_BMI | Self-reported pre-pregnancy body mass index | Numeric |
| Pregnancy complication | MM_FEVER^1^ | Fever? | 1 = YES  2 = NO |
|  | MM_NOMD^1^ | No medical risk factors? | 1 = YES  2 = NO |
|  | MM_PROM^1^ | Ruptured membrane? | 1 = YES  2 = NO |
| Health behavior | VITAMIN | Multivitamin – # of times/week | 1 = DIDN’T TAKE VITAMIN  2 = 1-3 TIMES/WEEK  3 = 4-6 TIMES/WEEK  4 = EVERY DAY/WEEK |
| Pregnancy intention | PGINTENT | Pregnancy intention | 1 = LATER  2 = SOONER  3 = THEN  4 = DID NOT WANT THEN OR ANY TIME  5 = WAS NOT SURE |
| Prenatal care | PNC_1TRM | Start prenatal care in the 1^st^ trimester? | 1 = YES  2 = NO  3 = NO PNC |
|  | PNC_VST_NAPHSIS^1^ | Number of prenatal care visits grouped | 1 = ≤ 8  2 = 9-11  3 = 12+ |
| Smoking | CIG_PRIOR^1^ | No. of cigarettes smoked prior to this pregnancy | Numeric |
|  | CIG_1TRI^1^ | No. of cigarettes smoked in the 1st trimester | Numeric |
|  | CIG_2TRI^1^ | No. of cigarettes smoked in the 2nd trimester | Numeric |
|  | CIG_3TRI^1^ | No. of cigarettes smoked in the 3rd trimester | Numeric |

*Note.*

^1^ The variable was extracted from the birth certificate data linked to the Pregnancy Risk Assessment Monitoring System data.

^2^ A newly created variable assigning “NO” when a participant reported no health insurance and “YES” when a participant had at least one type of health insurance, either private, public (Healthcare.gov, Medicaid, or state-specific option), or other.

**Table S3**

*Sample Characteristics by Maternal Race/Ethnicity among Unweighted Sample Populations*

|  | **Overall**  N = 78356^1^ | **N-H Black**  N = 17916^1^ | **N-H White**  N = 60440^1^ | ***p-*value**^2^ |
| --- | --- | --- | --- | --- |
| **Maternal Age** |  |  |  | <0.001 |
| <= 17 | 772 (1.0%) | 353 (2.0%) | 419 (0.7%) |  |
| 18-19 | 2746 (3.5%) | 894 (5.0%) | 1852 (3.1%) |  |
| 20-24 | 15436 (20%) | 4718 (26%) | 10718 (18%) |  |
| 25-29 | 24002 (31%) | 5169 (29%) | 18833 (31%) |  |
| 30-34 | 23149 (30%) | 4166 (23%) | 18983 (31%) |  |
| 35-39 | 10181 (13%) | 2092 (12%) | 8089 (13%) |  |
| 40+ | 2070 (2.6%) | 524 (2.9%) | 1546 (2.6%) |  |
| **Marital Status** |  |  |  | <0.001 |
| Married | 49976 (64%) | 5603 (31%) | 44373 (73%) |  |
| Not Married | 28380 (36%) | 12313 (69%) | 16067 (27%) |  |
| **Health Insurance Before Pregnancy** |  |  |  | <0.001 |
| Insured | 69092 (88%) | 15575 (87%) | 53517 (89%) |  |
| Uninsured | 9264 (12%) | 2341 (13%) | 6923 (11%) |  |
| **Total Annual Income^3^** |  |  |  | <0.001 |
| $0 to $15000 (Lv 01) | 15627 (20%) | 7142 (40%) | 8485 (14%) |  |
| $15001 to $19000 (Lv 02) | 5486 (7.0%) | 2023 (11%) | 3463 (5.7%) |  |
| $19001 to $22000 (Lv 03) | 4043 (5.2%) | 1414 (7.9%) | 2629 (4.3%) |  |
| $22001 to $26000 (Lv 04) | 3495 (4.5%) | 1112 (6.2%) | 2383 (3.9%) |  |
| $26001 to $29000 (Lv 05) | 2932 (3.7%) | 839 (4.7%) | 2093 (3.5%) |  |
| $29001 to $37000 (Lv 06) | 5075 (6.5%) | 1246 (7.0%) | 3829 (6.3%) |  |
| $37001 to $44000 (Lv 07) | 4279 (5.5%) | 838 (4.7%) | 3441 (5.7%) |  |
| $44001 to $52000 (Lv 08) | 4351 (5.6%) | 645 (3.6%) | 3706 (6.1%) |  |
| $52001 to $56000 (Lv 09) | 2534 (3.2%) | 345 (1.9%) | 2189 (3.6%) |  |
| $56001 to $67000 (Lv 10) | 4654 (5.9%) | 535 (3.0%) | 4119 (6.8%) |  |
| $67001 to $79000 (Lv 11) | 4828 (6.2%) | 450 (2.5%) | 4378 (7.2%) |  |
| $79001 or more (Lv 12) | 21052 (27%) | 1327 (7.4%) | 19725 (33%) |  |
| **No. of Household Members** |  |  |  | <0.001 |
| Mean (SD) | 2.93 (1.35) | 2.87 (1.54) | 2.94 (1.29) |  |
| **Maternal Education (Years)** |  |  |  | <0.001 |
| 00-08 | 634 (0.8%) | 211 (1.2%) | 423 (0.7%) |  |
| 09-11 | 5329 (6.8%) | 2073 (12%) | 3256 (5.4%) |  |
| 12 | 16829 (21%) | 5400 (30%) | 11429 (19%) |  |
| 13-15 | 25902 (33%) | 7028 (39%) | 18874 (31%) |  |
| 16+ | 29662 (38%) | 3204 (18%) | 26458 (44%) |  |
| **Receive WIC During Pregnancy** | 27881 (36%) | 11343 (63%) | 16538 (27%) | <0.001 |
| **Physical Abuse Before Pregnancy** | 2017 (2.6%) | 756 (4.2%) | 1261 (2.1%) | <0.001 |
| **Physical Abuse During Pregnancy** | 1697 (2.2%) | 701 (3.9%) | 996 (1.6%) | <0.001 |
| **Divorce** | 5251 (6.7%) | 2028 (11%) | 3223 (5.3%) | <0.001 |
| **Homeless** | 1974 (2.5%) | 962 (5.4%) | 1012 (1.7%) | <0.001 |
| **Job Loss of Husband/Partner** | 8521 (11%) | 2399 (13%) | 6122 (10%) | <0.001 |
| **Job Loss of Self** | 7631 (9.7%) | 3160 (18%) | 4471 (7.4%) | <0.001 |
| **Cut in Work Hours or Pay of Husband/Partner/Self** | 13619 (17%) | 3541 (20%) | 10078 (17%) | <0.001 |
| **Argument More Than Usual** | 17285 (22%) | 5982 (33%) | 11303 (19%) | <0.001 |
| **Unwanted Pregnancy by Husband/Partner** | 5612 (7.2%) | 2104 (12%) | 3508 (5.8%) | <0.001 |
| **Problem Paying Bill** | 15179 (19%) | 4552 (25%) | 10627 (18%) | <0.001 |
| **Imprisonment of Husband/Partner/Self** | 3179 (4.1%) | 1358 (7.6%) | 1821 (3.0%) | <0.001 |
| **Problem with Drinking/Drugs of People Close to Me** | 9938 (13%) | 2034 (11%) | 7904 (13%) | <0.001 |
| **Death of People Close to Me** | 14782 (19%) | 4218 (24%) | 10564 (17%) | <0.001 |
| **Depression Before Pregnancy** | 10099 (13%) | 1949 (11%) | 8150 (13%) | <0.001 |
| **Termination of Pregnancy** | 0.48 (0.95) | 0.63 (1.11) | 0.44 (0.90) | <0.001 |
| **Diabetes Before Pregnancy** | 2206 (2.8%) | 676 (3.8%) | 1530 (2.5%) | <0.001 |
| **Hypertension Before Pregnancy** | 4686 (6.0%) | 1807 (10%) | 2879 (4.8%) | <0.001 |
| **BMI Before Pregnancy** | 26.70 (6.85) | 28.55 (7.56) | 26.16 (6.52) | <0.001 |
| **Gestational Diabetes** | 6350 (8.1%) | 1748 (9.8%) | 4602 (7.6%) | <0.001 |
| **Fever During Pregnancy** | 1114 (1.4%) | 320 (1.8%) | 794 (1.3%) | <0.001 |
| **Medical Risks** |  |  |  | <0.001 |
| No Risks | 61748 (79%) | 13385 (75%) | 48363 (80%) |  |
| Risks | 16608 (21%) | 4531 (25%) | 12077 (20%) |  |
| **Premature Rupture of Membrane** | 4364 (5.6%) | 1039 (5.8%) | 3325 (5.5%) | 0.13 |
| **Intake of Multivitamin (Times/Week)** |  |  |  | <0.001 |
| 0 | 39373 (50%) | 11681 (65%) | 27692 (46%) |  |
| 1-3 | 5947 (7.6%) | 1538 (8.6%) | 4409 (7.3%) |  |
| 4-6 | 5389 (6.9%) | 802 (4.5%) | 4587 (7.6%) |  |
| 7 | 27647 (35%) | 3895 (22%) | 23752 (39%) |  |
| **Pregnancy Intention** |  |  |  | <0.001 |
| Later | 16533 (21%) | 5313 (30%) | 11220 (19%) |  |
| Not Sure | 11804 (15%) | 3854 (22%) | 7950 (13%) |  |
| Not Want | 5185 (6.6%) | 2107 (12%) | 3078 (5.1%) |  |
| Sooner | 11275 (14%) | 1615 (9.0%) | 9660 (16%) |  |
| Then | 33559 (43%) | 5027 (28%) | 28532 (47%) |  |
| **Start of PNC in 1st Trimester** |  |  |  | <0.001 |
| No | 9042 (12%) | 3378 (19%) | 5664 (9.4%) |  |
| No PNC | 412 (0.5%) | 141 (0.8%) | 271 (0.4%) |  |
| Yes | 68902 (88%) | 14397 (80%) | 54505 (90%) |  |
| **No. of PNC Visits** |  |  |  | <0.001 |
| <= 08 | 14994 (19%) | 4622 (26%) | 10372 (17%) |  |
| 09-11 | 24005 (31%) | 5363 (30%) | 18642 (31%) |  |
| 12+ | 39357 (50%) | 7931 (44%) | 31426 (52%) |  |
| **No. Cigarettes Before Pregnancy** | 1.86 (5.92) | 1.14 (4.61) | 2.08 (6.24) | <0.001 |
| **No. Cigarettes in 1st Trimester** | 1.16 (4.33) | 0.70 (3.22) | 1.30 (4.60) | <0.001 |
| **No. Cigarettes in 2nd Trimester** | 0.86 (3.53) | 0.49 (2.65) | 0.98 (3.75) | <0.001 |
| **No. Cigarettes in 3rd Trimester** | 0.76 (3.30) | 0.42 (2.49) | 0.86 (3.49) | <0.001 |
| **Gestational Age** |  |  |  | <0.001 |
| <= 27 | 1323 (1.7%) | 435 (2.4%) | 888 (1.5%) |  |
| 28-33 | 3702 (4.7%) | 911 (5.1%) | 2791 (4.6%) |  |
| 34-36 | 7453 (9.5%) | 1779 (9.9%) | 5674 (9.4%) |  |
| 37+ | 65878 (84%) | 14791 (83%) | 51087 (85%) |  |
| **Preterm Birth** |  |  |  |  |
| Yes | 12478 (16%) | 3125 (17%) | 9353 (15%) | <0.001 |

*Note.*

PNC = prenatal care, SD = standard deviation, WIC = Special Supplemental Nutrition Program for Women, Infants, and Children.

^1^ *n* (%) for categorical variables and mean (*SD*) for continuous variables.

^2^ Pearson’s Chi-squared tests (for categorical variables) and Wilcoxon rank sum tests (for continuous variables) were conducted.

^3^ The total incomes shown in the table indicate values only from the Phase 7 data. The Phase 8 data have different values (slightly higher than those from Phase 7) in each category after taking the inflation into account. However, both Phases have 12 income categories, which were entered into the models as income tiers. The adjusted amount of income under each category from Phase 8 can be found in Table S2 above.

**Table S4**

*Sample Characteristics by Maternal Race/Ethnicity among Weighted (Replicated) Sample Populations*

|  | **Overall**  N = 5004138^1^ | **N-H Black**  N = 739801^1^ | **N-H White**  N = 4264337^1^ | ***p*-value**^2^ |
| --- | --- | --- | --- | --- |
| **Maternal Age** |  |  |  | <0.001 |
| <= 17 | 31671 (0.6%) | 9538 (1.3%) | 22133 (0.5%) |  |
| 18-19 | 142419 (2.8%) | 31511 (4.3%) | 110908 (2.6%) |  |
| 20-24 | 917893 (18%) | 195987 (26%) | 721906 (17%) |  |
| 25-29 | 1552223 (31%) | 224136 (30%) | 1328087 (31%) |  |
| 30-34 | 1543052 (31%) | 171771 (23%) | 1371281 (32%) |  |
| 35-39 | 684708 (14%) | 86316 (12%) | 598392 (14%) |  |
| 40+ | 132172 (2.6%) | 20542 (2.8%) | 111630 (2.6%) |  |
| **Marital Status** |  |  |  | <0.001 |
| Married | 3362670 (67%) | 230001 (31%) | 3132669 (73%) |  |
| Not Married | 1641468 (33%) | 509800 (69%) | 1131668 (27%) |  |
| **Health Insurance Before Pregnancy** |  |  |  | <0.001 |
| Insured | 4450887 (89%) | 633436 (86%) | 3817451 (90%) |  |
| Uninsured | 553251 (11%) | 106365 (14%) | 446886 (10%) |  |
| **Health Insurance During Pregnancy** |  |  |  | <0.001 |
| Insured | 4,919,884 (98%) | 728,810 (99%) | 4,191,074 (98%) |  |
| Uninsured | 84,254 (1.7%) | 10,991 (1.5%) | 73,263 (1.7%) |  |
| **Total Annual Income^3^** |  |  |  | <0.001 |
| $0 to $15000 (Lv 01) | 820577 (16%) | 281152 (38%) | 539425 (13%) |  |
| $15001 to $19000 (Lv 02) | 319959 (6.4%) | 82958 (11%) | 237001 (5.6%) |  |
| $19001 to $22000 (Lv 03) | 231071 (4.6%) | 57317 (7.7%) | 173754 (4.1%) |  |
| $22001 to $26000 (Lv 04) | 204450 (4.1%) | 44176 (6.0%) | 160274 (3.8%) |  |
| $26001 to $29000 (Lv 05) | 179644 (3.6%) | 36323 (4.9%) | 143321 (3.4%) |  |
| $29001 to $37000 (Lv 06) | 307451 (6.1%) | 51029 (6.9%) | 256422 (6.0%) |  |
| $37001 to $44000 (Lv 07) | 269176 (5.4%) | 34264 (4.6%) | 234912 (5.5%) |  |
| $44001 to $52000 (Lv 08) | 284032 (5.7%) | 29420 (4.0%) | 254612 (6.0%) |  |
| $52001 to $56000 (Lv 09) | 162646 (3.3%) | 14566 (2.0%) | 148080 (3.5%) |  |
| $56001 to $67000 (Lv 10) | 309061 (6.2%) | 24688 (3.3%) | 284373 (6.7%) |  |
| $67001 to $79000 (Lv 11) | 329011 (6.6%) | 20359 (2.8%) | 308652 (7.2%) |  |
| $79001 or more (Lv 12) | 1587060 (32%) | 63549 (8.6%) | 1523511 (36%) |  |
| **No. of Household Members** | 2.93 (1.30) | 2.82 (1.50) | 2.95 (1.27) | <0.001 |
| **Maternal Education (Years)** |  |  |  | <0.001 |
| 00-08 | 39075 (0.8%) | 5823 (0.8%) | 33252 (0.8%) |  |
| 09-11 | 285724 (5.7%) | 79683 (11%) | 206041 (4.8%) |  |
| 12 | 1024378 (20%) | 223960 (30%) | 800418 (19%) |  |
| 13-15 | 1544553 (31%) | 284370 (38%) | 1260183 (30%) |  |
| 16+ | 2110408 (42%) | 145965 (20%) | 1964443 (46%) |  |
| **Receive WIC During Pregnancy** | 1577115 (32%) | 457880 (62%) | 1119235 (26%) | <0.001 |
| **Physical Abuse Before Pregnancy** | 103889 (2.1%) | 27712 (3.7%) | 76177 (1.8%) | <0.001 |
| **Physical Abuse During Pregnancy** | 86783 (1.7%) | 24947 (3.4%) | 61836 (1.5%) | <0.001 |
| **Divorce** | 288061 (5.8%) | 83329 (11%) | 204732 (4.8%) | <0.001 |
| **Homeless** | 100595 (2.0%) | 36576 (4.9%) | 64019 (1.5%) | <0.001 |
| **Job Loss of Husband/Partner** | 504782 (10%) | 103499 (14%) | 401283 (9.4%) | <0.001 |
| **Job Loss of Self** | 432970 (8.7%) | 134984 (18%) | 297986 (7.0%) | <0.001 |
| **Cut in Work Hours or Pay of Husband/Partner/Self** | 828668 (17%) | 153616 (21%) | 675052 (16%) | <0.001 |
| **Argument More Than Usual** | 1013422 (20%) | 247888 (34%) | 765534 (18%) | <0.001 |
| **Unwanted Pregnancy by Husband/Partner** | 314224 (6.3%) | 85398 (12%) | 228826 (5.4%) | <0.001 |
| **Problem Paying Bill** | 891129 (18%) | 192495 (26%) | 698634 (16%) | <0.001 |
| **Imprisonment of Husband/Partner/Self** | 159200 (3.2%) | 46969 (6.3%) | 112231 (2.6%) | <0.001 |
| **Problem with Drinking/Drugs of People Close to Me** | 598080 (12%) | 77482 (10%) | 520598 (12%) | <0.001 |
| **Death of People Close to Me** | 885192 (18%) | 164713 (22%) | 720479 (17%) | <0.001 |
| **Depression Before Pregnancy** | 595770 (12%) | 67349 (9.1%) | 528421 (12%) | <0.001 |
| **Termination of Pregnancy** | 0.46 (0.91) | 0.64 (1.11) | 0.43 (0.86) | <0.001 |
| **Diabetes Before Pregnancy** | 118930 (2.4%) | 21449 (2.9%) | 97481 (2.3%) | <0.001 |
| **Hypertension Before Pregnancy** | 233303 (4.7%) | 62698 (8.5%) | 170605 (4.0%) | <0.001 |
| **BMI Before Pregnancy** | 26.45 (6.59) | 28.44 (7.37) | 26.1 (6.38) | <0.001 |
| **Gestational Diabetes** | 387384 (7.7%) | 68943 (9.3%) | 318441 (7.5%) | <0.001 |
| **Fever During Pregnancy** | 69469 (1.4%) | 12674 (1.7%) | 56795 (1.3%) | <0.001 |
| **Medical Risks** |  |  |  | <0.001 |
| No Risks | 4130561 (83%) | 578547 (78%) | 3552014 (83%) |  |
| Risks | 873577 (17%) | 161254 (22%) | 712323 (17%) |  |
| **Premature Rupture of Membrane** | 211508 (4.2%) | 34827 (4.7%) | 176681 (4.1%) | <0.001 |
| **Intake of Multivitamin (Times/Week)** |  |  |  | <0.001 |
| 0 | 2408660 (48%) | 485349 (66%) | 1923311 (45%) |  |
| 1-3 | 376748 (7.5%) | 63232 (8.5%) | 313516 (7.4%) |  |
| 4-6 | 365906 (7.3%) | 33253 (4.5%) | 332653 (7.8%) |  |
| 7 | 1852824 (37%) | 157967 (21%) | 1694857 (40%) |  |
| **Pregnancy Intention** |  |  |  | <0.001 |
| Later | 1008919 (20%) | 223861 (30%) | 785058 (18%) |  |
| Not Sure | 698269 (14%) | 155061 (21%) | 543208 (13%) |  |
| Not Want | 290421 (5.8%) | 87215 (12%) | 203206 (4.8%) |  |
| Sooner | 751467 (15%) | 64873 (8.8%) | 686594 (16%) |  |
| Then | 2255062 (45%) | 208791 (28%) | 2046271 (48%) |  |
| **Start of PNC in 1st Trimester** |  |  |  | <0.001 |
| No | 529522 (11%) | 138638 (19%) | 390884 (9.2%) |  |
| No PNC | 6194 (0.1%) | 981 (0.1%) | 5213 (0.1%) |  |
| Yes | 4468422 (89%) | 600182 (81%) | 3868240 (91%) |  |
| **No. of PNC Visits** |  |  |  | <0.001 |
| <= 08 | 738126 (15%) | 180481 (24%) | 557645 (13%) |  |
| 09-11 | 1545588 (31%) | 227831 (31%) | 1317757 (31%) |  |
| 12+ | 2720424 (54%) | 331489 (45%) | 2388935 (56%) |  |
| **No. Cigarettes Before Pregnancy** | 1.8 (5.8) | 1.0 (4.4) | 1.9 (6.0) | <0.001 |
| **No. Cigarettes in 1st Trimester** | 1.0 (4.1) | 0.6 (2.8) | 1.1 (4.3) | <0.001 |
| **No. Cigarettes in 2nd Trimester** | 0.77 (3.39) | 0.39 (2.21) | 0.83 (3.55) | <0.001 |
| **No. Cigarettes in 3rd Trimester** | 0.68 (3.15) | 0.33 (2.01) | 0.74 (3.31) | <0.001 |
| **Gestational Age** |  |  |  | <0.001 |
| <= 27 | 19122 (0.4%) | 7818 (1.1%) | 11304 (0.3%) |  |
| 28-33 | 63728 (1.3%) | 17683 (2.4%) | 46045 (1.1%) |  |
| 34-36 | 263482 (5.3%) | 51795 (7.0%) | 211687 (5.0%) |  |
| 37+ | 4657806 (93%) | 662505 (90%) | 3995301 (94%) |  |
| **Preterm Birth** | 346332 (6.9%) | 77296 (10%) | 269036 (6.3%) | <0.001 |

*Note.*

PNC = prenatal care, WIC = Special Supplemental Nutrition Program for Women, Infants, and Children.

^1^ n (%); mean (SD)

^2^ Pearson’s Chi-squared test; Wilcoxon rank sum test

^3^ The total incomes shown in the table indicate values only from the Phase 7 data. The Phase 8 data have different values (slightly higher than those from Phase 7) in each category after taking the inflation into account. However, both Phases have 12 income categories, which were entered into the models as income tiers. The adjusted amount of income under each category from Phase 8 can be found in Table S2 above.

**Table S5**

*Number and Rates of Preterm Birth by Maternal Characteristics among Unweighted Sample Populations*

|  | **Overall**  N = 12478^1,2^ | **N-H Black**  N = 3125^1,2^ | **N-H White**  N = 9353^1,2^ |
| --- | --- | --- | --- |
| **Maternal Age** |  |  |  |
| <= 17 | 135 (17%) | 52 (15%) | 83 (20%) |
| 18-19 | 505 (18%) | 138 (15%) | 367 (20%) |
| 20-24 | 2544 (16%) | 764 (16%) | 1780 (17%) |
| 25-29 | 3650 (15%) | 837 (16%) | 2813 (15%) |
| 30-34 | 3455 (15%) | 760 (18%) | 2695 (14%) |
| 35-39 | 1746 (17%) | 444 (21%) | 1302 (16%) |
| 40+ | 443 (21%) | 130 (25%) | 313 (20%) |
| **Marital Status** |  |  |  |
| Married | 7218 (14%) | 929 (17%) | 6289 (14%) |
| Not Married | 5260 (19%) | 2196 (18%) | 3064 (19%) |
| **Health Insurance Before Pregnancy** |  |  |  |
| Insured | 10772 (16%) | 2659 (17%) | 8113 (15%) |
| Uninsured | 1706 (18%) | 466 (20%) | 1240 (18%) |
| **Total Annual Income^3^** |  |  |  |
| $0 to $15000 (Lv 01) | 2934 (19%) | 1225 (17%) | 1709 (20%) |
| $15001 to $19000 (Lv 02) | 997 (18%) | 349 (17%) | 648 (19%) |
| $19001 to $22000 (Lv 03) | 753 (19%) | 248 (18%) | 505 (19%) |
| $22001 to $26000 (Lv 04) | 614 (18%) | 189 (17%) | 425 (18%) |
| $26001 to $29000 (Lv 05) | 497 (17%) | 151 (18%) | 346 (17%) |
| $29001 to $37000 (Lv 06) | 832 (16%) | 236 (19%) | 596 (16%) |
| $37001 to $44000 (Lv 07) | 642 (15%) | 138 (16%) | 504 (15%) |
| $44001 to $52000 (Lv 08) | 664 (15%) | 106 (16%) | 558 (15%) |
| $52001 to $56000 (Lv 09) | 386 (15%) | 65 (19%) | 321 (15%) |
| $56001 to $67000 (Lv 10) | 664 (14%) | 97 (18%) | 567 (14%) |
| $67001 to $79000 (Lv 11) | 669 (14%) | 91 (20%) | 578 (13%) |
| $79001 or more (Lv 12) | 2826 (13%) | 230 (17%) | 2596 (13%) |
| **No. of Household Members** | 2.84 (1.38) | 2.80 (1.57) | 2.86 (1.30) |
| **Maternal Education (Years)^3^** |  |  |  |
| 00-08 | 122 (19%) | 38 (18%) | 84 (20%) |
| 09-11 | 1055 (20%) | 378 (18%) | 677 (21%) |
| 12 | 3046 (18%) | 932 (17%) | 2114 (18%) |
| 13-15 | 4266 (16%) | 1239 (18%) | 3027 (16%) |
| 16+ | 3989 (13%) | 538 (17%) | 3451 (13%) |
| **Receive WIC During Pregnancy** | 4816 (17%) | 1835 (16%) | 2981 (18%) |
| **Physical Abuse Before Pregnancy^3^** | 416 (21%) | 131 (17%) | 285 (23%) |
| **Physical Abuse During Pregnancy^3^** | 337 (20%) | 124 (18%) | 213 (21%) |
| **Divorce** | 1076 (20%) | 391 (19%) | 685 (21%) |
| **Homeless^1^** | 373 (19%) | 147 (15%) | 226 (22%) |
| **Job Loss of Husband/Partner^3^** | 1539 (18%) | 423 (18%) | 1116 (18%) |
| **Job Loss of Self^3^** | 1466 (19%) | 569 (18%) | 897 (20%) |
| **Cut in Work Hours or Pay of Husband/Partner/Self^3^** | 2331 (17%) | 621 (18%) | 1710 (17%) |
| **Argument More Than Usual^3^** | 2941 (17%) | 1033 (17%) | 1908 (17%) |
| **Unwanted Pregnancy by Husband/Partner^3^** | 1046 (19%) | 384 (18%) | 662 (19%) |
| **Problem Paying Bill^3^** | 2769 (18%) | 823 (18%) | 1946 (18%) |
| **Imprisonment of Husband/Partner/Self^3^** | 618 (19%) | 234 (17%) | 384 (21%) |
| **Problem with Drinking/Drugs of People Close to Me^3^** | 1797 (18%) | 359 (18%) | 1438 (18%) |
| **Death of People Close to Me^3^** | 2600 (18%) | 750 (18%) | 1850 (18%) |
| **Depression Before Pregnancy^3^** | 2009 (20%) | 361 (19%) | 1648 (20%) |
| **Termination of Pregnancy** | 0.59 (1.13) | 0.80 (1.35) | 0.52 (1.04) |
| **Diabetes Before Pregnancy** | 579 (26%) | 192 (28%) | 387 (25%) |
| **Hypertension Before Pregnancy** | 1386 (30%) | 558 (31%) | 828 (29%) |
| **BMI Before Pregnancy** | 27.32 (7.45) | 28.99 (7.90) | 26.77 (7.21) |
| **Gestational Diabetes** | 1278 (20%) | 360 (21%) | 918 (20%) |
| **Fever During Pregnancy** | 245 (22%) | 91 (28%) | 154 (19%) |
| **Medical Risk Factors** |  |  |  |
| No Risks | 7610 (12%) | 1752 (13%) | 5858 (12%) |
| Risks | 4868 (29%) | 1373 (30%) | 3495 (29%) |
| **Premature Rupture of Membrane** | 2313 (53%) | 596 (57%) | 1717 (52%) |
| **Intake of Multivitamin (Times/Week)** |  |  |  |
| 0 | 6556 (17%) | 2013 (17%) | 4543 (16%) |
| 1-3 | 831 (14%) | 261 (17%) | 570 (13%) |
| 4-6 | 667 (12%) | 115 (14%) | 552 (12%) |
| 7 | 4424 (16%) | 736 (19%) | 3688 (16%) |
| **Pregnancy Intention** |  |  |  |
| Later | 2685 (16%) | 871 (16%) | 1814 (16%) |
| Not Sure | 2048 (17%) | 687 (18%) | 1361 (17%) |
| Not Want | 949 (18%) | 374 (18%) | 575 (19%) |
| Sooner | 1898 (17%) | 339 (21%) | 1559 (16%) |
| Then | 4898 (15%) | 854 (17%) | 4044 (14%) |
| **Start of PNC in 1st Trimester** |  |  |  |
| No | 1,522 (17%) | 591 (17%) | 931 (16%) |
| No PNC | 125 (30%) | 46 (33%) | 19 (29%) |
| Yes | 10831 (16%) | 2488 (17%) | 8343 (15%) |
| **No. of PNC Visits** |  |  |  |
| <= 08 | 5899 (39%) | 1632 (35%) | 4267 (41%) |
| 09-11 | 3545 (15%) | 780 (15%) | 2765 (15%) |
| 12+ | 3034 (7.7%) | 713 (9.0%) | 2321 (7.4%) |
| **No. Cigarettes Before Pregnancy** | 2.55 (6.87) | 1.49 (5.34) | 2.90 (7.28) |
| **No. Cigarettes in 1st Trimester** | 1.7 (5.3) | 0.92 (3.70) | 1.96 (5.72) |
| **No. Cigarettes in 2nd Trimester** | 1.29 (4.29) | 0.64 (2.91) | 1.51 (4.64) |
| **No. Cigarettes in 3rd Trimester** | 1.11 (4.04) | 0.57 (3.15) | 1.29 (4.28) |

*Note.*

PNC = prenatal care, WIC = Special Supplemental Nutrition Program for Women, Infants, and Children.

^1^ n (%); mean (SD)

^2^ Pearson’s Chi-squared test; Wilcoxon rank sum test

^3^ There was no statistically significant association between preterm birth and total annual income (*p* = 0.8), maternal education (*p* = 0.7), physical abuse by a husband/partner before (*p* > 0.9) and during pregnancy (*p* = 0.9), homeless (*p* = 0.07), job loss of husband/partner (*p* = 0.8), job loss of self (*p* = 0.4), cut in work hours or pay of husband/partner/self (*p* = 0.9), argument more than usual (*p* = 0.7), unwanted pregnancy by husband/partner (*p* = 0.3), problem paying bill (*p* = 0.2), imprisonment of husband/partner/self (*p* = 0.8), problem with drinking/drugs of people close to me (*p* = 0.8), death of people close to me (*p* = 0.5), and depression before pregnancy (*p* = 0.2) among N-H Black women.

**Table S6**

*Number and Rates of Preterm Birth by Maternal Characteristics among Weighted (Replicated) Sample Populations*

|  | Overall  N = 5004138^1,2^ | N-H Black  N = 739801^1,2^ | N-H White  N = 4264337^1,2^ |
| --- | --- | --- | --- |
| **Maternal Age** |  |  |  |
| <= 17 | 2648 (8.4%) | 624 (6.5%) | 2024 (9.1%) |
| 18-19 | 12724 (8.9%) | 3579 (11.4%) | 9145 (8.3%) |
| 20-24 | 62493 (6.8%) | 18259 (9.3%) | 44234 (6.1%) |
| 25-29 | 104233 (6.7%) | 22553 (10.1%) | 81680 (6.2%) |
| 30-34 | 101893 (6.6%) | 19143 (11.1%) | 82750 (6.0%) |
| 35-39 | 49456 (7.2%) | 10398 (12.0%) | 39058 (6.5%) |
| 40+ | 12885 (9.8%) | 2740 (13.3%) | 10145 (9.1%) |
| **Marital Status** |  |  |  |
| Married | 205752 (6.1%) | 22526 (9.8%) | 183226 (5.9%) |
| Not Married | 140580 (8.6%) | 54770 (10.7%) | 85810 (7.6%) |
| **Health Insurance Before Pregnancy** |  |  |  |
| Insured | 299955 (6.7%) | 65945 (10.4%) | 234010 (6.1%) |
| Uninsured | 46377 (8.4%) | 11351 (10.7%) | 35026 (7.8%) |
| **Health Insurance During Pregnancy** |  |  |  |
| Insured | 340,246 (6.9%) | 75,924 (10%) | 264,322 (6.3%) |
| Uninsured | 6,086 (7.2%) | 1,372 (12%) | 4,714 (6.4%)^4^ |
| **Total Annual Income^3^** |  |  |  |
| $0 to $15,000 (Lv 01) | 76048 (9.3%) | 32013 (11.4%) | 44035 (8.2%) |
| $15,001 to $19,000 (Lv 02) | 26586 (8.3%) | 9144 (11.0%) | 17442 (7.4%) |
| $19,001 to $22,000 (Lv 03) | 18164 (7.9%) | 5009 (8.7%) | 13155 (7.6%) |
| $22,001 to $26,000 (Lv 04) | 15814 (7.7%) | 4136 (9.4%) | 11678 (7.3%) |
| $26,001 to $29,000 (Lv 05) | 12157 (6.8%) | 3523 (9.7%) | 8634 (6.0%) |
| $29,001 to $37,000 (Lv 06) | 22010 (7.2%) | 5343 (10.5%) | 16667 (6.5%) |
| $37,001 to $44,000 (Lv 07) | 18553 (6.9%) | 2712 (7.9%) | 15841 (6.7%) |
| $44,001 to $52,000 (Lv 08) | 17924 (6.3%) | 3034 (10.3%) | 14890 (5.9%) |
| $52,001 to $56,000 (Lv 09) | 9643 (5.9%) | 1608 (11.0%) | 8035 (5.4%) |
| $56,001 to $67,000 (Lv 10) | 20352 (6.6%) | 2565 (10.4%) | 17787 (6.3%) |
| $67,001 to $79,000 (Lv 11) | 17994 (5.5%) | 2623 (12.9%) | 15371 (5.0%) |
| $79,001 or more (Lv 12) | 91087 (5.7%) | 5586 (8.8%) | 85501 (5.6%) |
| **No. of Household Members** | 2.90 (1.38) | 2.87 (1.68) | 2.90 (1.29) |
| **Maternal Education (Years)** |  |  |  |
| 00-08 | 2835 (7.3%) | 602 (10.3%) | 2233 (6.7%) |
| 09-11 | 27827 (9.7%) | 9884 (12.4%) | 17943 (8.7%) |
| 12 | 84929 (8.3%) | 24863 (11.1%) | 60066 (7.5%) |
| 13-15 | 111191 (7.2%) | 29528 (10.4%) | 81663 (6.5%) |
| 16+ | 119550 (5.7%) | 12419 (8.5%) | 107131 (5.5%) |
| **Receive WIC During Pregnancy** |  |  |  |
| Yes | 128757 (8.2%) | 46954 (10.3%) | 81803 (7.3%) |
| No | 217575 (6.4%) | 30342 (10.8%) | 187233 (6.0%) |
| **Physical Abuse Before Pregnancy** | 9924 (9.6%) | 2721 (9.8%) | 7203 (9.5%) |
| **Physical Abuse During Pregnancy** | 7887 (9.1%) | 2675 (10.7%)^4^ | 5212 (8.4%) |
| **Divorce** | 28829 (10%) | 9866 (11.8%) | 18963 (9.3%) |
| **Homeless** | 9698 (9.6%) | 3490 (9.5%) | 6208 (9.7%) |
| **Job Loss of Husband/Partner** | 39384 (7.8%) | 10805 (10.4%)^4^ | 28579 (7.1%) |
| **Job Loss of Self** | 41190 (9.5%) | 15866 (11.8%) | 25324 (8.5%) |
| **Cut in Work Hours or Pay of Husband/Partner/Self** | 65108 (7.9%) | 16658 (10.8%) | 48450 (7.2%) |
| **Argument More Than Usual** | 78736 (7.8%) | 25170 (10.2%) | 53566 (7.0%) |
| **Unwanted Pregnancy by Husband/Partner** | 24035 (7.7%) | 9557 (11.2%) | 14478 (6.3%)^4^ |
| **Problem Paying Bill** | 72483 (8.1%) | 21869 (11.4%) | 50614 (7.2%) |
| **Imprisonment of Husband/Partner/Self** | 16710 (10.5%) | 4852 (10.3%)^4^ | 11858 (10.6%) |
| **Problem with Drinking/Drugs of People Close to Me** | 46661 (7.8%) | 8849 (11.4%) | 37812 (7.3%) |
| **Death of People Close to Me** | 69468 (7.9%) | 18595 (11.3%) | 50873 (7.1%) |
| **Depression Before Pregnancy** | 53122 (8.9%) | 9039 (13.4%) | 44083 (8.3%) |
| **Termination of Pregnancy** | 0.57 (1.06) | 0.81 (1.36) | 0.50 (0.95) |
| **Diabetes Before Pregnancy** | 15896 (13.4%) | 3759 (17.5%) | 12137 (12.5%) |
| **Hypertension Before Pregnancy** | 33772 (14.5%) | 12300 (19.6%) | 21472 (12.6%) |
| **BMI Before Pregnancy** | 27.52 (7.41) | 29.07 (8.02) | 27.07 (7.17) |
| **Gestational Diabetes** | 38748 (10%) | 7811 (11.3%) | 30937 (9.7%) |
| **Fever During Pregnancy** | 5653 (8.1%) | 1771 (14.0%) | 3882 (6.8%) |
| **Medical Risk Factors** | 127258 (14.6%) | 30851 (19.1%) | 96407 (13.5%) |
| **Premature Rupture of Membrane** | 57833 (27.3%) | 14201 (40.8%) | 43632 (24.7%) |
| **Intake of Multivitamin (Times/Week)** |  |  |  |
| 0 | 175586 (7.3%) | 50518 (10.4%) | 125068 (6.5%) |
| 1-3 | 23815 (6.3%) | 6203 (9.8%) | 17612 (5.6%) |
| 4-6 | 19866 (5.4%) | 2592 (7.8%) | 17274 (5.2%) |
| 7 | 127065 (6.9%) | 17983 (11.4%) | 109082 (6.4%) |
| **Pregnancy Intention** |  |  |  |
| Later | 73270 (7.3%) | 22390 (10%) | 50880 (6.5%) |
| Not Sure | 55119 (7.9%) | 16144 (10.4%) | 38975 (7.2%) |
| Not Want | 25097 (8.6%) | 9621 (11%) | 15476 (7.6%) |
| Sooner | 53833 (7.2%) | 7807 (12%) | 46026 (6.7%) |
| Then | 139013 (6.2%) | 21334 (10.2%) | 117679 (5.8%) |
| **Start of PNC in 1st Trimester** |  |  |  |
| No | 41814 (7.9%) | 15690 (11.3%) | 26124 (6.7%) |
| No PNC | 1017 (16.4%) | 375 (38.2%) | 642 (12.3%) |
| Yes | 303501 (6.8%) | 61231 (10.2%) | 242270 (6.3%) |
| **No. of PNC Visits** |  |  |  |
| <= 08 | 134970 (18.3%) | 36795 (20.4%) | 98175 (17.6%) |
| 09-11 | 111353 (7.2%) | 18738 (8.2%) | 92615 (7.0%) |
| 12+ | 100009 (3.7%) | 21763 (6.6%) | 78246 (3.3%) |
| **No. Cigarettes Before Pregnancy** | 2.11 (6.03) | 1.38 (4.93) | 2.32 (6.29) |
| **No. Cigarettes in 1st Trimester** | 1.4 (4.7) | 0.82 (3.30) | 1.50 (5.04) |
| **No. Cigarettes in 2nd Trimester** | 1.04 (3.76) | 0.61 (2.72) | 1.17 (4.00) |
| **No. Cigarettes in 3rd Trimester** | 0.90 (3.46) | 0.49 (2.42) | 1.02 (3.70) |

*Note.*

PNC = prenatal care, WIC = Special Supplemental Nutrition Program for Women, Infants, and Children.

^1^ n (%); mean (SD)

^2^ Pearson’s Chi-squared test; Wilcoxon rank sum test

^3^ The total incomes shown in the table indicate values only from the Phase 7 data. The Phase 8 data have different values (slightly higher than those from Phase 7) in each category after taking the inflation into account. However, both Phases have 12 income categories, which were entered into the models as income tiers. The adjusted amount of income under each category from Phase 8 can be found in Table S2 above.

^4^ The difference from the counterpart was not statistically significant.

**Table S7**

*Comparison of Prediction Accuracy between Prior and Post Model Calibration*

|  | Train | |  | Test | | | |
| --- | --- | --- | --- | --- | --- | --- | --- |
|  | Not Calibrated | Calibrated |  | Not Calibrated | | Calibrated | |
| AUC/Brier^1^ | All | All |  | Term | PTB | Term | PTB |
|  | Unweighted | | | | | | |
| Pooled^2^ | 0.811/0.102 | 0.811/0.102 |  | 0.813/0.103 | 0.187/0.695 | 0.813/0.103 | 0.187/0.695 |
| N-H Black^2^ | 0.806/0.111 | 0.806/0.112 |  | 0.799/0.111 | 0.201/0.678 | 0.799/0.111 | 0.201/0.669 |
| N-H White^3^ | 0.810/0.100 | 0.810/0.100 |  | 0.814/0.099 | 0.186/0.700 | 0.814/0.099 | 0.186/0.699 |
|  | Weighted (Replicated) | | | | | | |
| Pooled^4^ | 0.758/0.058 | 0.759/0.058 |  | 0.760/0.058 | 0.240/0.827 | 0.760/0.058 | 0.240/0.822 |
| N-H Black^3^ | 0.756/0.0779 | 0.756/0.0778 |  | 0.754/0.078 | 0.246/0.765 | 0.754/0.078 | 0.246/0.765 |
| N-H White^4^ | 0.763/0.0536 | 0.764/0.0536 |  | 0.765/0.0536 | 0.235/0.839 | 0.766/0.0536 | 0.234/0.833 |

*Note.*

^1^ Mean of the out-of-fold estimates (k = 5)

^2^ Beta calibration was conducted.

^3^ Logistic regression calibration was conducted.

^4^ Isotonic calibration was conducted.

**Figure S1**

*Feature Importance in Preterm Birth Risk Prediction among Non-Hispanic Black Women (Unweighted Data)*

*
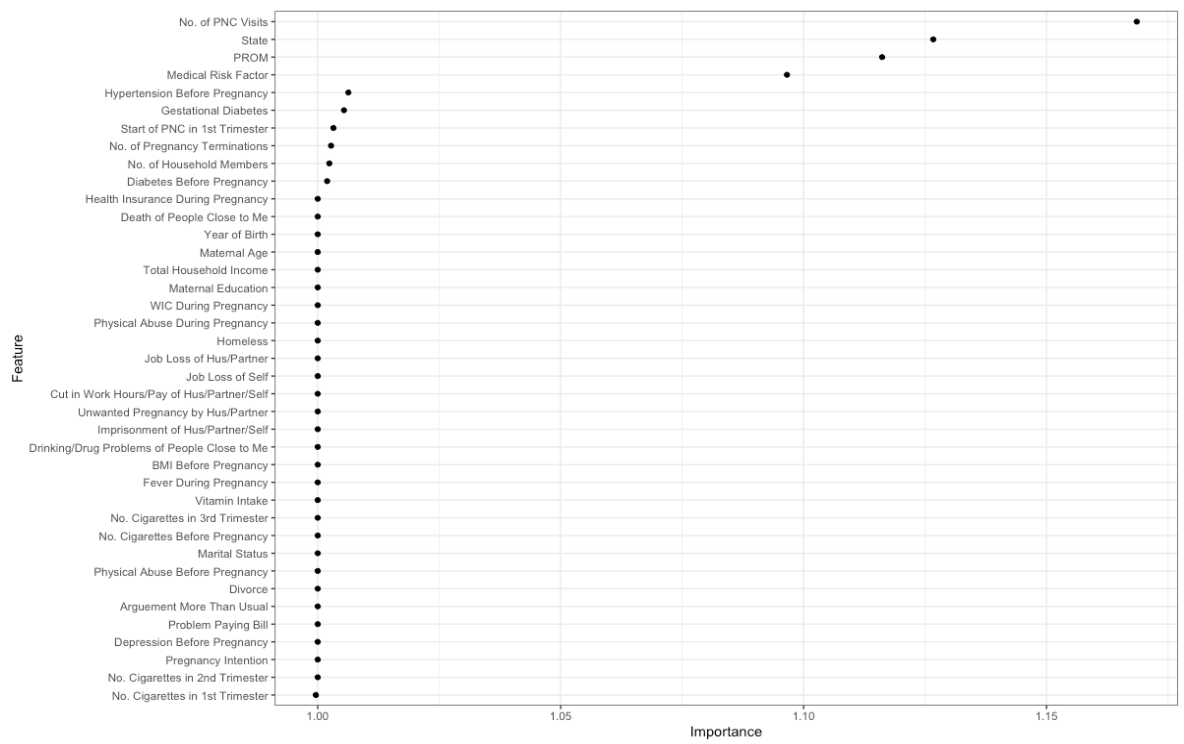
*

**Figure S2**

*Feature Importance in Preterm Birth Risk Prediction among Non-Hispanic White Women (Unweighted Data)*

*
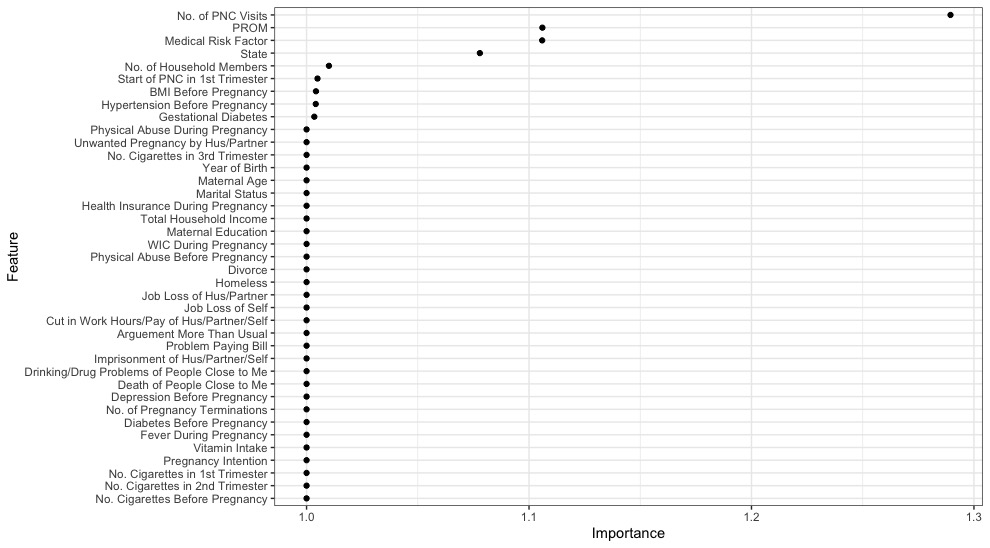
*

**Figure S3**

*Effect of the Important Feature on Predicted Probability of Preterm Birth among Non-Hispanic Black Women (Weighted/Replicated Data)*

*
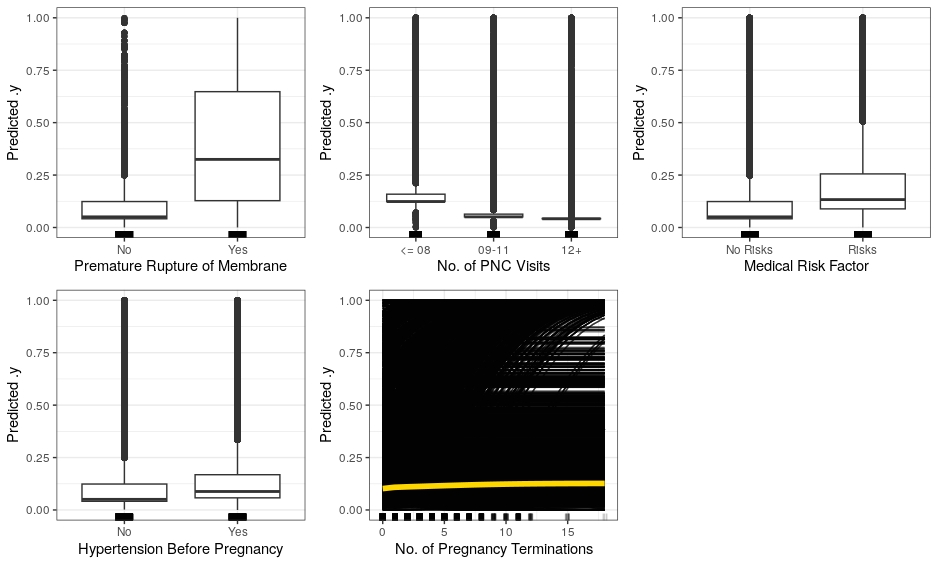
*

**Figure S4**

*Effect of the Important Feature on Predicted Probability of Preterm Birth among Non-Hispanic White Women (Weighted/Replicated Data)*


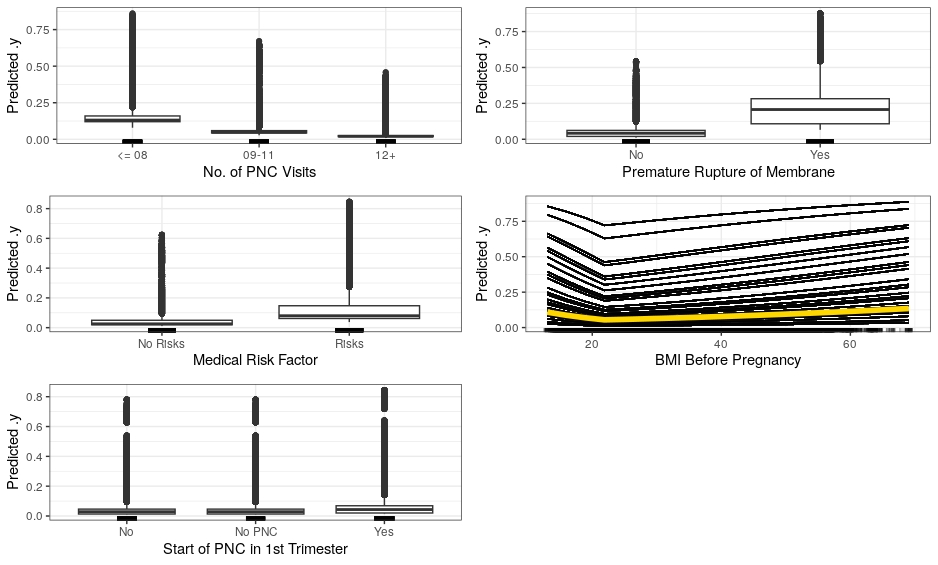

Supplement: Supplementary file 1 — Supplementary Material 1 [file 12884_2024_6613_MOESM1_ESM.docx]
